# Validation of SSR markers for identification of high-yielding and *Phytophthora Capsici* root rot resistant chilli genotypes

**DOI:** 10.1038/s41598-024-79718-z

**Published:** 2024-11-19

**Authors:** Tazien Bukhari, Rashid Mehmood Rana, Azeem Iqbal Khan, Muhammad Azam Khan, Atta Ullah, Misbah Naseem, Humaira Rizwana, Mohamed S. Elshikh, Muhammad Rizwan, Rashid Iqbal

**Affiliations:** 1 Department of Plant Breeding and Genetics, PMAS-Arid Agriculture University, Rawalpindi, 46300 Pakistan; 2 Department of Plant Breeding and Genetics, University of Agriculture Faisalabad, Faisalabad, 03802 Pakistan; 3 Department of Horticulture, PMAS-Arid Agriculture University, Rawalpindi, 46300 Pakistan; 4 Department of Plant Pathology, Agriculture Research Institute, Mingora, Swat, 19200 Pakistan; 5 Department of Botany and Microbiology, College of Science, King Saud University, P.O. 2455, Riyadh, 11451 Saudi Arabia; 6 Institute of Crop Science and Resource Conservation (INRES), University of Bonn, 53115 Bonn, Germany; 7 Department of Agronomy, Faculty of Agriculture and Environment, The Islamia University of Bahawalpur, Bahawalpur, 63100 Pakistan; 8 Department of Life Sciences, Western Caspian University, Baku, Azerbaijan

**Keywords:** *Phytophthora capsici*, Genomics, Simple sequence repeats, Marker assisted breeding, Root Rot, Biological techniques, Biotechnology, Plant sciences

## Abstract

The study was designed to validate the previously reported 34 SSR markers using 78 chilli genotypes to detect significant trait specific markers as well as superior genotypes resistant to *Phytophthora capsici* root rot (PcRR). In this context, the identification of germplasm with higher yield per plant (YPP) leads to hype in stress tolerance index (STI) in genotypes, Chakwal3 (11.98), Greenfire (10.14), Advanta5017 (9.94) and Chakwal4 (7.8). The identified genotypes were also found as resistant and moderately resistant due to existence of below 50% of disease incidence. Moreover, biplot showed the interrelation of STI with YPP through the formation of acute angle by their respective vectors. In the current study, the markers Hpms1172 and CAMS177 was found significant for STI. However, the marker CAMS066 was found associated with relative cell injury, CA06g27450 with disease incidence and CAMS173 with relative leaf damage. The bright bands on gel pictures of significant markers showed the association of these markers with resistant genotypes i.e. Chakwal3, Advanta-5017 and Chakwal4 as well as with a single moderately resistant genotype i.e. Greenfire. The markerstudes confirmed the phenotypic data by showing association of markers i.e. Hpms1172 and CAMS177, r with stress tolerance index. The principal coordinate analysis aligned with the results obtained from marker-assisted selection. Thus, currently practiced marker assisted selection detected high yielding genotypes in PcRR disease stress condition that will be helpful in progressing breeding programs in chilli.

**Supplementary Information:**

The online version contains supplementary material available at 10.1038/s41598-024-79718-z.

## Introduction

Chilli is a versatile vegetable crop used extensively as spice, pharmaceutical product, ornamental plant, ingredient in cosmetics, weapon, and biopesticide^1^. Globally, China is the largest producer of green chilli, while India leads in dry chilli production. Worldwide, green chilli is grown on 2.0 Mha (million hectare) of area while dry chilli is on 1.6 Mha of area. Worldwide production of green and dry chilli is 36.2 MT (million tons) and 4.8 MT with the yield of 17.6 and 2.9 tons per hactare, respectively^2^. Globally Pakistan stands among the top ten producers of dry chilli^2^. According to the economic survey of Pakistan 2022-23, chilli crop is cultivated under 0.03 Mha of area with 0.08 MT of production that shares 1.5% in country ‘s GDP (Gross domestic product)^3^.

Chilli, as one of the most highly demanded condiment, has inspired chilli breeders worldwide to continually develop and improve chilli varieties for increased yield. However, chilli yield is often hindered by biotic and abiotic factors. Among biotic stresses, the root rot caused by *Phytophthora capsici*, has become a serious limitation in chilli production around the world, often leading to complete yield loss^1^. It is the 5th highly destructive oomycete that once entered into soil can survive more than 10–20 years due to its broad host range, having dormant and motile zoospores and is difficult to control through cultural practices^4^. The causal organism *Phytophthora capsici*, flourishes on wet soil with 80–100% moisture, on stagnant water, and on 20–30 °C of temperature^5^. An attack by *Phytophthora* on chilli plants leads to damping-off lesions at basal part of the stems, which eventually cause stem and root girdling, resulting in the sudden wilting of leaves^6^. Invasion of pathogens triggers the excessive production of reactive oxygen species (ROS) through mitochondria, chloroplasts, and peroxisomes of host plant, causing damage to the plant’s macromolecules^7^. Initially, ROS serves as an antimicrobial signal in response to the pathogen-host interaction, known as oxidative burst^8,9^. Latterly, ROS damages the cell membrane, disrupts redox balance that leads to the production of malondialdehyde and lipid peroxides. This process contributes to membrane damage and programmed cell death (PCD) that ultimately resulting in appearance of necrosis spots on host cells^10,11^.

The management of *Phytophthora capsici* is challenging because of its broad host range, soil-borne characteristics, and unpredictable mating habits. Moreover, its control is complicated due to the existence of 45 physiological races of *Phytophthora*, which require distinct R genes including Snakin-1^12^, CaPhyto^13^, CaDMR1^14^ etc. Resistance in *Capsicum* to *Phytophthora capsici* root rot (PcRR) is genetically and physiologically complex with reports of single, two and multiple gene system being involved^15^. It is necessary to develop strategies to control PcRR and to understand the genetics of plant-pathogen interaction. Therefore, developing varieties carrying effective resistance against destructive pathogens has become a priority for breeders. This varietal development can be attained through phenotypic and genotypic characterization of crop.

The reliability of phenotypic screening is often questioned due to the significant influence of environmental factors, which lead to unstable and false positive selections^16,17^. Therefore, the use of molecular markers is proved as a powerful alternative that helps in perfection of genotypic screening due to its environment free impact. Resistance to Phytophthora in chilli has previously been assessed through the identification of molecular markers, such as SSR markers: CAMS 405, Hpms 1–62, CAMS 839, HpmsE034 etc., CAPS markers: ASC037, ASC031 and SCAR marker: ASC035p^18–20^. The validation of tightly linked molecular markers using marker assisted selection (MAS) method is the prerequisites for the successful selection of resistant genotype^21^. The marker assisted selection was previously reported by Alsaleh et al., where they detected wheat genotypes having low Cadmium content^22^. Due to the limited number of existing studies, this research was designed to identify strongly associated markers by validating 34 previously detected SSR markers and to select high-yielding, disease-resistant genotypes using a marker-assisted selection (MAS) approach for Phytophthora capsici root rot (PcRR) resistance in chilli^23^. The identified markers will facilitate in the transfer of disease-resistant genes in superior chilli genotypes, but these will also aid in identification of *Phytophthora* resistant genotypes.

## Results

### Phenotypic assessment of chilli germplasm

The seventy-eight chilli genotypes were assessed using data obtained from characterization of eight phenotypic traits viz. YPPc; yield per plant under the control condition (g), YPPi; yield per plant under the infected condition (g), STI; stress tolerance index, RCI; relative cell injury (%), CV; cell viability (%), DI; disease incidence (%), DSI; disease severity index (%) and RLD; relative leaf damage (%) for PcRR resistance. The obtained data were further subjected to ANOVA and principal component analysis (PCA).

### Yield per plant

The analysis of variance for yield per plant displays the significance in both conditions (Table 1). Moreover, YPPc was ranged from 2.10 g (15/4) to 98.61 g (Chakwal3) with mean value of 26 g, however, YPPi was observed with 1.76 g (15/4) to 90.69 g (Chakwal3) range of values, with mean of 24 g value (Table 1). Furthermore, the mean performance of chilli genotypes was measured by comparing adjusted means of genotypes with least significant increase (LSI) based means of checks (Supplementary Table S5 online). The LSI based mean value of superior check Chakwal3 depicted the maximum yield per plant under control condition (mean _check_ +LSI _check_=99.01 g) when compared to the adjusted values of other chilli germplasm. Similarly, on comparison with adjusted values of chilli genotypes, the superior check Chakwal3 was found best performing for yield per plant under the infected condition (mean _check_+LSI _check_=90.70 g).

**Table 1:** The analysis of variance and descriptive statistics for seventy-eight Chilli genotypes.

	Df	YPPc	YPPi	STI	RCI	CV	DI	DSI	RLD
**Block unadjusted**	3	1895	1246	19.8	276.63	700.97	390.98	733.2	383.01
**Genotypes adjusted**	77	610**	533**	10.8**	402.55**	563.87***	963.11**	1183**	232.11**
**Control**	1	1585**	2472**	34.9**	1027.3**	442.98 ***	262.89**	6194.4**	509.16**
**Control vs. augmented**	76	597**	507**	10.4**	396.76**	565.46***	972.32**	1117.1**	228.46**
**Residuals**	3	17	9	0.12	75.62	0.37	0.32	0.8	0.78
**Coefficient of variation**		16%	12%	3.1%	13.9%	1.2%	0.7%	2%	6%
**Range**		2.10-98.61	1.76–90.69	0.01–11.98	23.16–88.56	16.11–93.96	6.81–100	1.12–100	1.10-89.45
**Means**		26	24	2.2	66.06	52.34	78.66	44.77	15.22
**Least significant increase**		19.34	10.24	0.14	3.41	0.42	0.36	0.91	0.89

YPPc(g) = yield per plant under control condition; YPPi(g) = yield per plant under infected condition; STI = stress tolerance index; RCI = relative cell injury; CV(%) = cell viability; DI(%) = disease incidence; DSI(%) = disease severity index; RLD(%) = relative leaf damage;

Significance codes:‘**= significant; NS = non-significant.

### Stress tolerance index

The analysis of variance was observed with significant mean sum of square for stress tolerance index is shown in Table 1. The range of STI values were observed from 0.01 (16163) to 11.98 (Chakwal3) with mean value of 2.2. Moreover, the comparison of adjusted values of chilli genotypes with LSI based value of checks found that best performing check Chakwal3 has maximum STI (mean _check_+LSI _check_ =12.12) than other genotypes (Supplementary Table S5 online).

### Relative cell injury

The Table 1 shows the analysis of variance for relative cell injury, that has the significant mean sum of square. Moreover, the minimum RCI was observed in genotype Greenfire with 23.16% whereas Advanta512 was found with maximum cell injury i.e. 88.56% with mean of 66.06% value (Fig. 2a). Plants detected with low relative cell injury under stress resulted in higher yield and termed as best performer. Therefore, based on LSI test, adjusted values of all genotypes exceeded from superior check Chakwal3 that confirmed the presence of lowest RCI (mean _check_+LSI _check_=28.11%) in Chakwal3 (Supplementary Table S5 online).

**Fig. 1:**
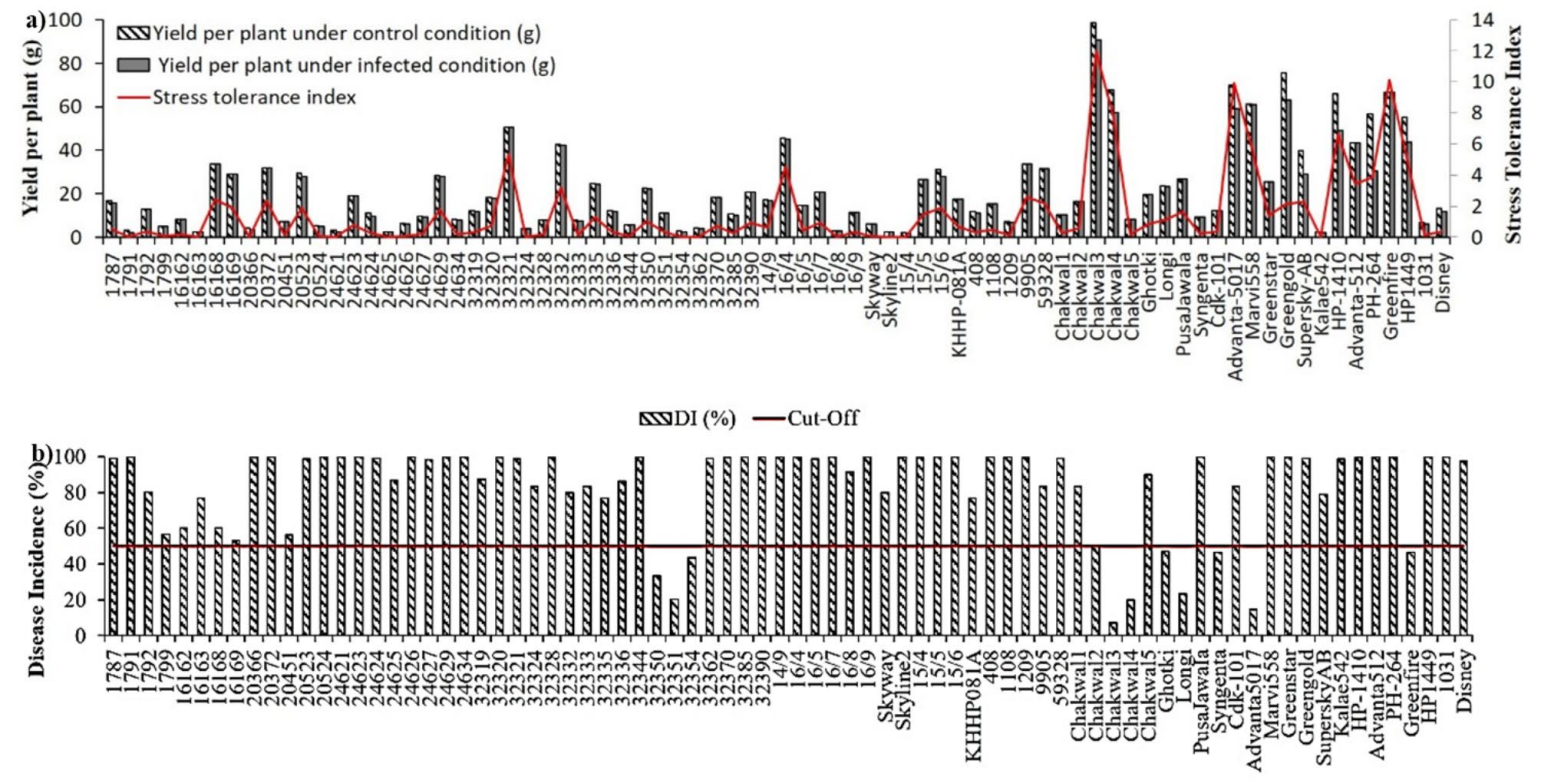
**(a)** Bar graphs are depicting yield per plant-YPP (g) in the control and in the infected conditions, however, red line is showing stress tolerance index-STI for *Phytophthora capsici* root rot in chilli and **(b)** is showing disease incidence-DI (%) based on resistance level scale whereas red line is showing the threshold level (50%) between resistance and susceptibility for *Phytophthora capsici* root rot in chilli.

**Fig. 2:**
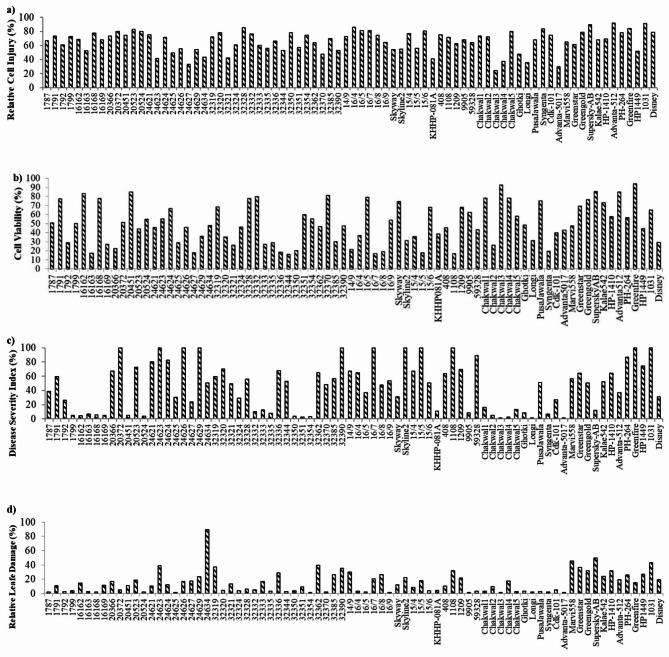
**(a)** Relative cell injury-RCI (%), **(b)** cell viability-CV (%), **(c)** disease severity index-DSI (%) and **(d)** relative leaf damage-RLD (%) for *Phytophthora capsici* root rot in chilli.

### Cell viability

The analysis of variance in Table 1, depicted the significant mean sum of square for cell viability. The highest CV of 93.96% was recorded for genotype Greenfire whereas 32,344 was found with minimum CV i.e. 16.11% with mean value of 52.34% (Fig. 2b). The LSI based value of checks revealed that the adjusted value of genotype Greenfire (94.17%) was exceeded from superior check Chakwal3 (mean _check_+LSI _check_=93.31%) (Supplementary Table S5 online).

### Disease incidence

The analysis of variance was observed with significant mean sum of square for disease incidence is shown in Table 1. Moreover, the minimum DI of 6.81% was shown in genotype Chakwal3 whereas twenty-four numbers of genotypes were found with maximum DI of 100% with mean value of 78.66% (Table 1). A plant with reduced disease incidence percentage was considered to be healthier. Therefore, based on LSI test the check Chakwal3 was found superior with lowest DI (mean _check_+LSI _check_=7.17%), then adjusted values of all chilli genotypes (Supplementary Table S5 online).

### Disease severity index

The analysis of variance with significant mean sum of square for disease severity index (%) is shown in Table 1. The genotype Chakwal3 was found with maximum DSI i.e. 1.12% whereas four genotypes i.e. 24,629, 32,390, Skyline 2 and 1108, were observed with 100% DSI with mean value of 44.77% (Table 1; Fig. 2c). Plants detected with low DSI under stress condition considered as healthier plants. Therefore, the comparison of LSI based mean of checks with adjusted values of all genotypes detected that genotype Advanta5017 was found better for DSI trait (1.82%) than superior check Chakwal3 (mean _check_+LSI _check_=2.01%) (Supplementary Table S5 online).

### Relative leaf damage

The analysis of variance has shown the significant mean sum of square for relative leaf damage (%) that is given in Table 1. RLD value was observed ranges from 1.10% (32354) to 89.45% (24634) with mean value of 15.22% (Table 1 Fig. 2d). Low percentage of RLD in plants under stress resulted in healthier plants. Therefore, the adjusted values of four genotypes i.e. 32,354 (0.57%), Advanta5017 (0.57%), 24,625 (0.67%) and Longi (0.74%) exceeded from the LSI based mean value of superior check Chakwal3 for trait RLD (mean _check_+LSI _check_=0.90%) (Supplementary Table S5 online).

### Selection of resistant germplasm

The resistance level scale classified 3 genotypes (Chakwal3, Advanta5017 and Chakwal4) as resistant: R, followed by 7 genotypes (32351, Longi, 32350, 32354, Syngenta, Ghotki and Greenfire) as moderately resistant: MR and the rest of 68 genotypes as susceptible: S (Fig. 1-b, Supplementary Table S6 online).

### Principal component analysis (PCA)

**Fig. 3:**
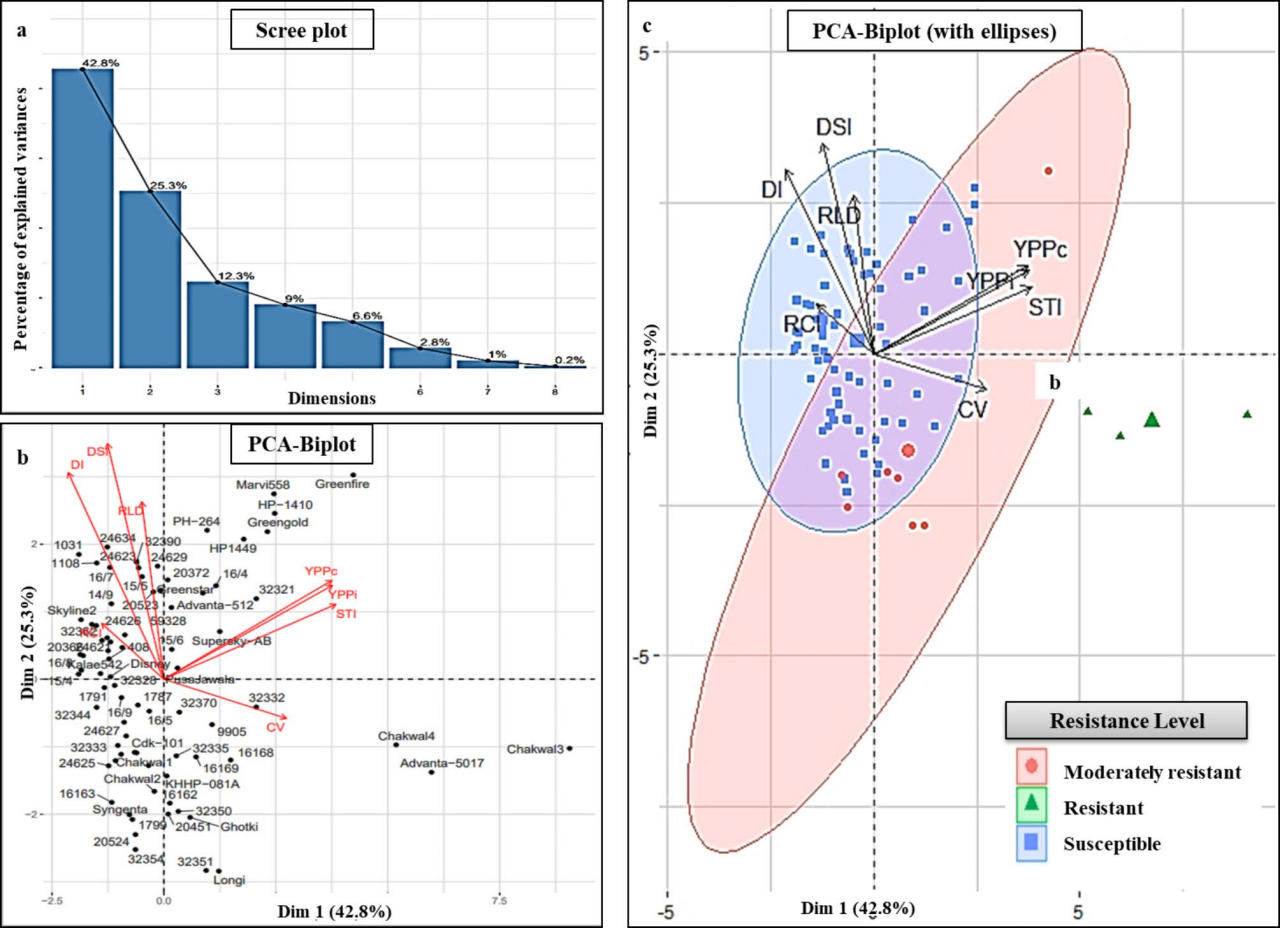
Scree plot **(a)**, Biplot **(b)**, showing distribution of genotypes and traits whereas biplot **(c)**, showing ellipses for three resistance levels in chilli germplasm.

The data of phenotypic traits were subjected to principal component analysis (PCA) that resulted with maximum variance for PC1 and PC2 (42.8% and 25.30%, respectively) contributing 68.08% of cumulative variance (Supplementary Table S6 online) as evident from scree plot (Fig. 3-a). The traits having component loading value of 0.5 or more are considered as major contributors. Therefore, the major contribution was observed by STI with 0.86 value for PC1 whereas DI for PC2 with 0.71 value is given in Supplementary Table S6. The PCA biplot (Fig. 3-b) was generated on basis of first two principal components to detect the significant chilli genotypes and the relationship among all measured traits. The biplot (Fig. 3-b) showed that genotypes Chakwal3, Chakwal4, Advanta5017, and Greenfire were found far from the central point that depicts the significance of these genotypes in the current germplasm collection. Conversely, the vectors on PCA biplot with acute angle show positive correlation, obtuse angle show negative correlation while right angle shows zero correlation among trait. Therefore, the biplot depicts the close correlation of traits RLD, DI, DSI and RLD and are oppositely correlated with traits YPPc, YPPi, STI and CV (Fig. 3-c and -b). The Fig. 3-c shows the presence of genotypes in their respective ellipses: Resistant, moderately resistant and susceptible on basis of resistance level scale (Supplementary Table S5 online). It clearly separated resistant genotypes from moderately resistant and susceptible genotypes. Saturation of genotypes was found in ellipses for susceptible genotypes.

### Molecular assessment

The previously detected thirty-four PcRR associated SSR markers^22^ were amplified for seventy-eight chilli genotypes in order to find significant SSRs that leads to successful marker assisted selection.

### Validation of markers

**Fig. 4:**
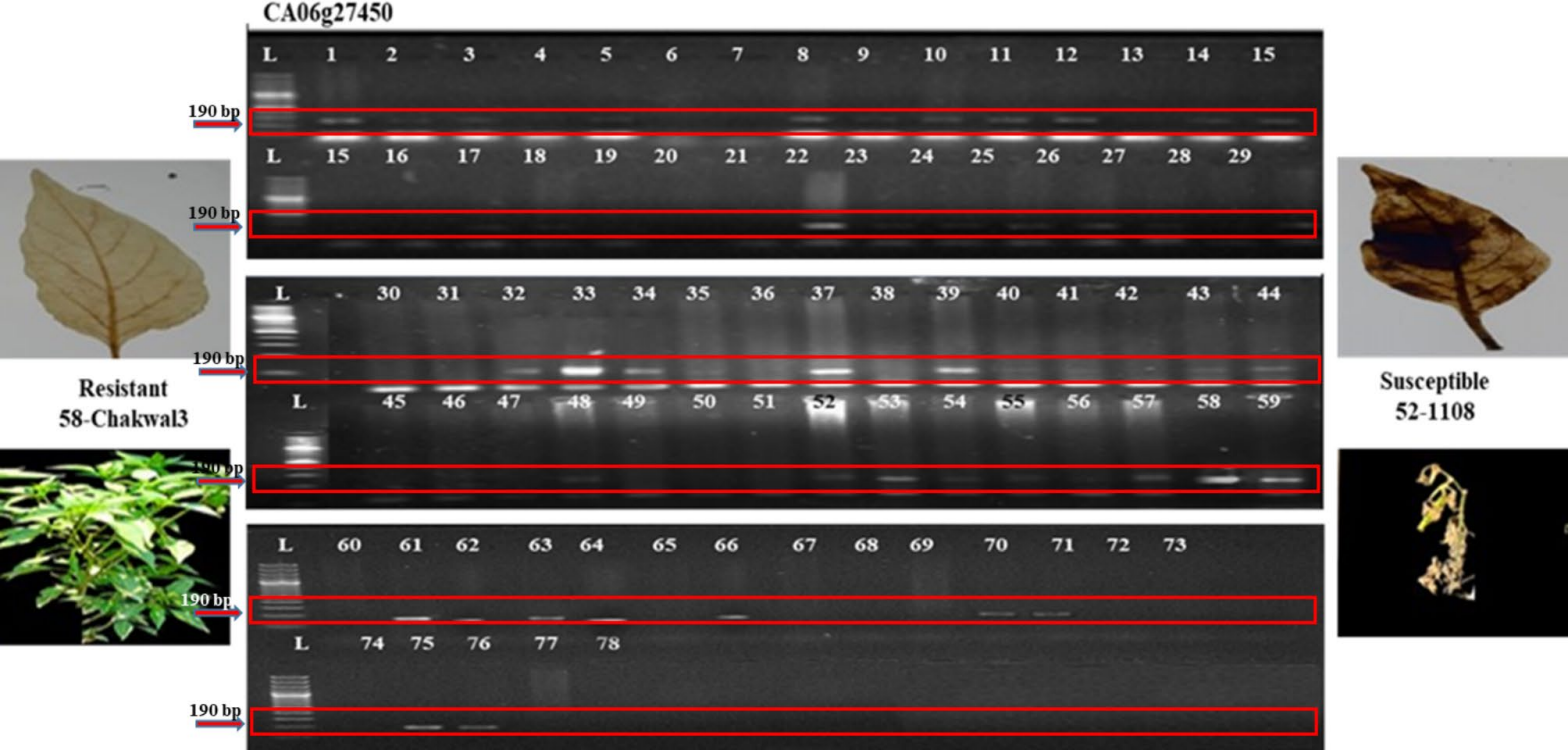
The gel picture of significant marker CA06g27450 associated with disease incidence showing visualization of bands for resistant and susceptible chilli genotypes along with pixels of their respective plants and H_2_O_2_ accumulated DAB stained leaves.

The means for traits of 78 genotypes among 34 SSR markers were grouped by comparing them on basis of binary data i.e. 1 for presence and 0 for absence of the marker using student’s *t*-test. The student’s *t*-test detected five significant trait specific markers out of thirty-four markers. The SSR markers. Hpms1172 and CAMS177 were found significant for stress tolerance index with 0.0003 and 0.0465 P-value, respectively. Whereas, marker CAMS066 with 0.0435 P-value for relative cell injury, CA06g27450 with 0.0003 P-value for disease incidence and CAMS173 with 0.0001 P-value for relative leaf damage, were found significant (Table 2). The Fig. 4 is the gel picture of marker CA06g27450 found significant for disease incidence shows the presence of bright band for resistant genotype i.e. Chakwal3 as well as absence of bands for susceptible chilli genotype i.e. 1108 (Table 2). Moreover, the Table 3 showed that maximum genotypes were found associated for markers CAMS177, CAMS066, CA06g27450 and CAMS173 whereas eight genotypes were found associated with marker Hpms1172.

**Table 2:** List of validated SSR markers along with their associated phenotypic traits.

Markers	*P*-value	Associated traits
Hpms1172	0.0003*	Stress tolerance index
CAMS177	0.0465*	Stress tolerance index
CAMS066	0.0435*	Relative cell injury
CA06g27450	0.0003*	Disease incidence
CAMS173	0.0001*	Relative leaf damage
*=significant		

**Table 3:** Amplification of Chilli genotypes at the 5 validated SSR markers by showing ‘+’ sign for the presence of bands whereas ‘–‘ sign for the absence.

S#	Genotype	Hpms1172	CAMS177	CAMS066	CA06g27450	CAMS173	S#	Genotype	Hpms1172	CAMS177	CAMS066	CA06g27450	CAMS173
	Size (bp)	200	150	290	190	210		Size (bp)	200	150	290	190	210
1	1787	-	-	-	-	-	41	16/5	-	-	-	-	-
**2**	**1791**	**-**	**-**	**-**	**-**	**-**	**42**	**16/7**	**-**	**-**	**-**	**-**	**-**
**3**	**1792**	**-**	**-**	**-**	**-**	**-**	**43**	**16/8**	**-**	**-**	**-**	**-**	**-**
**4**	**1799**	**-**	**-**	**-**	**-**	**-**	**44**	**16/9**	**-**	**-**	**-**	**-**	**-**
**5**	**16,162**	**-**	**-**	**-**	**-**	**-**	**45**	**Skyway**	**-**	**-**	**-**	**-**	**-**
**6**	**16,163**	**-**	**-**	**-**	**-**	**-**	**46**	**Skyline2**	**-**	**-**	**-**	**-**	**-**
**7**	**16,168**	**-**	**-**	**-**	**-**	**-**	**47**	**15/4**	**-**	**-**	**-**	**-**	**-**
**8**	**16,169**	**-**	**-**	**-**	**-**	**-**	**48**	**15/5**	**-**	**-**	**-**	**-**	**-**
**9**	**20,366**	**-**	**-**	**-**	**-**	**-**	**49**	**15/6**	**-**	**-**	**-**	**-**	**-**
**10**	**20,372**	**-**	**-**	**-**	**-**	**-**	**50**	**KHHP081A**	**-**	**-**	**-**	**-**	**-**
**11**	**20,451**	**-**	**-**	**-**	**-**	**-**	**51**	**408**	**-**	**-**	**-**	**-**	**-**
**12**	**20,523**	**-**	**-**	**-**	**-**	**-**	**52**	**1108**	**-**	**-**	**-**	**-**	**-**
**13**	**20,524**	**-**	**-**	**-**	**-**	**-**	**53**	**1209**	**-**	**-**	**-**	**-**	**-**
**14**	**24,621**	**-**	**-**	**-**	**-**	**-**	**54**	**9905**	**-**	**-**	**-**	**-**	**-**
**15**	**24,623**	**-**	**-**	**-**	**-**	**-**	**55**	**59,328**	**-**	**-**	**-**	**-**	**-**
**16**	**24,624**	**-**	**-**	**-**	**-**	**-**	**56**	**Chakwal1**	-	-	-	-	-
**17**	**24,625**	**-**	**-**	**-**	**-**	**-**	**57**	**Chakwal2**	-	-	-	-	-
**18**	**24,626**	**-**	**-**	**-**	**-**	**-**	**58**	**Chakwal3**	+	+	+	+	+
**19**	**24,627**	**-**	**-**	**-**	**-**	**-**	**59**	**Chakwal4**	+	+	+	+	+
**20**	**24,629**	**-**	**-**	**-**	**-**	**-**	**60**	**Chakwal5**	-	-	-	-	-
**21**	**24,634**	**-**	**-**	**-**	**-**	**-**	**61**	**Ghotki**	-	-	+	+	+
**22**	**32,319**	**-**	**-**	**-**	**-**	**-**	**62**	**Longi**	-	-	+	+	+
**23**	**32,320**	-	-	-	-	-	**63**	**PusaJawala**	-	-	-	-	-
**24**	**32,321**	+	+	-	-	-	**64**	**Syngenta**	-	-	+	+	+
**25**	**32,324**	-	-	-	-	-	**65**	**Cdk-101**	-	-	-	-	-
**26**	**32,328**	-	-	-	-	-	**66**	**Advanta5017**	+	+	+	+	+
**27**	**32,332**	-	-	-	-	-	**67**	**Marvi558**	-	+	-	-	-
**28**	**32,333**	-	-	-	-	-	**68**	**Greenstar**	-	-	-	-	-
**29**	**32,335**	-	-	-	-	-	**69**	**Greengold**	+	+	-	-	-
**30**	**32,336**	-	-	-	-	-	**70**	**Supersky-AB**	-	-	-	-	-
**31**	**32,344**	-	-	-	-	-	**71**	**Kalae542**	-	-	-	-	-
**32**	**32,350**	-	-	+	+	+	**72**	**HP-1410**	+	+	-	-	-
**33**	**32,351**	-	-	+	+	+	**73**	**Advanta-512**	-	-	-	-	-
**34**	**32,354**	-	-	+	+	+	**74**	**PH-264**	-	-	-	-	-
**35**	**32,362**	-	-	-	-	-	**75**	**Greenfire**	+	+	+	+	+
**36**	**32,370**	-	-	-	-	-	**76**	**HP1449**	-	+	-	-	-
**37**	**32,385**	-	-	-	-	-	**77**	**1031**	-	-	-	-	-
**38**	**32,390**	-	-	-	-	-	**78**	**Disney**	**-**	**-**	**-**	**-**	**-**
**39**	**14/9**	-	-	-	-	-	**Total present**		08	10	10	10	10
**40**	**16/4**	+	+	-	-	-	**Total absent**		70	68	68	68	68

### Marker assisted selection (MAS)

The significant trait specific markers were further leads to marker assisted selection by appearance of bright bands on the gel pixels for chilli genotypes (Supplementary Figure. S1a-e online). The gel picture of marker Hpms1172 has produced clear bands at 200 bp size for genotypes, Chakwal3, Greenfire, Greengold, Advanta5017, Chakwal4, 32,321, HP1410 and 16/4 (Supplementary Figure. S1-a online), whereas marker CAMS177 has shown clear bands at 150 bp size for genotypes, Chakwal3, Greenfire, Greengold, Advanta5017, Chakwal4, 32,321, HP1410, 16/4, Marvi558 and HP1449 (Supplementary Figure. S1-b online). Moreover, the gel picture of SSR markers viz. CAMS066, CA06g27450 and CAMS173 showed the appearance of bright bands at 290 bp, 190 bp and 210 bp of size, respectively for chilli genotypes, Chakwal3, Chakwal4, Longi, Advanta5017, 32,350, 32,351, 32,354, Ghotki, Syngenta and Greenfire (Supplementary Figure. S1c-e online).

**Fig. 5:**
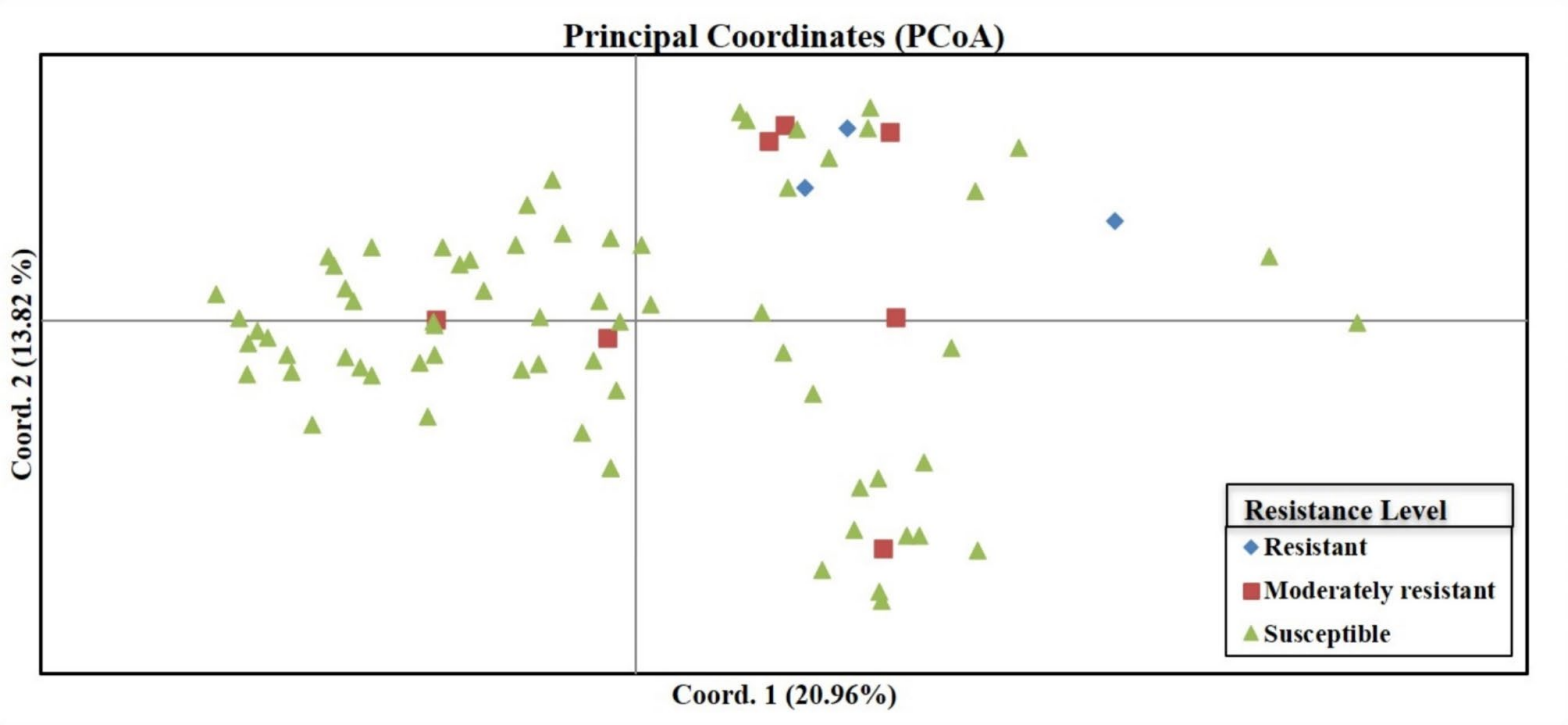
Principal coordinate analysis (PCoA) of chilli germplasm based on data of SSR markers.

Moreover, principal coordinate analysis (PCoA) was carried out to further validate the obtained data for MAS. Principal coordinate analysis (PCoA) plot confirmed the genetic relatedness and discrete grouping that yielded into significantly distinct populations (resistant, moderately resistant and susceptible) on Fig. 5. The first coordinate has shown 20.96% whereas second coordinate explained 13.82% of the variation with 34.78% of cumulative variance for both coordinates. The major contribution was observed by genotype Chakwal3 for both PCoA 1 and PCoA 2 with 0.97 and 0.48 values, respectively (Supplementary Table S7 online).

## Discussion

*Phytophthora capsici* root rot (PcRR) is one of the disastrous oomycetes, which causes drastic reduction in yield and productivity of chilli^24^. It is considered as a notorious pathogen that could cause damages from 30–100%^25^. Despite of the destructive nature of the disease, it is not controllable due to cultural practices. The fungicides are mostly used on infected fields for *Phytophthora* root rot management that resulted in development of fungicide resistance in crop that leads to environmental hazards. Moreover, presence of different *Phytophthora* races severely complicates the control of this pathogen. It requires keen attention of plant breeders to screen and detect PcRR resistant chilli genotypes which are phenotypically superior and are able to achieve sustainable production. Therefore, present study was designed for marker assisted selection and validation of previously reported 34 SSR markers using 78 chilli genotypes^23^.

A series of experiments were conducted to achieve the planned tasks to screen out PcRR resistant chilli genotypes having high yield tendency from a germplasm collection under stress (PcRR infected sick bed)^26^. Crop yield is the main target of plant breeders to improve the productivity and quality of their crop under stress^27,28^. Phenotypic assessment was majorly dependent on the screening of germplasm for disease resistance^29^. The superior genotypes with ability of disease resistance along with high yield were identified by selecting genotypes with maximum stress tolerance index^30,31^. Therefore, in current study the genotype Chakwal3 was found as best due to existence of maximum yield under both control:98.61 g and infected condition:90.69 g resulted in maximum stress tolerance index:11.98 value (Fig. 1-a; Supplementary Table S4 online). Thus, stress tolerance index is mostly used to differentiate between resistant and susceptible genotypes^32,33^. Moreover, the genotypes Greenfire, Advanta-5017 and Chakwal4 were also found best due to increased STI of 10.14, 9.94 and 7.8 value, respectively. It was noted that there was lack of significant findings in the quest to identify superior genotypes through stress tolerance index analysis in chilli, for both biotic and abiotic stresses. The STI was obtained in the study of Aktar-Uz-Zaman et al., to get desirable genotypes for high temperature condition in lentil crop, whereas, in the study of Mudi, Mahapatra, & Das, stable genotypes were identified for the assessment of *Helminthosporium* blight resistance in barley^30,34^.

Plants that exhibit resistant to disease have low percentage of disease incidence, which results in improvement of its health and growth. This improvement is supported by a healthy root system that enhances plant resistance, ultimately resulting in increased crop yield^35^. Therefore, the genotype Chakwal3 have shown reduced disease incidence (6.81%) and disease severity index (1.12%). Whereas, the genotype Greenfire was observed with minimum relative cell injury (23.16%) and maximum cell viability (93.96%). Our currently obtained results contribute more to useful physiological features in chilli that have been connected to PcRR resistance, which can be used as the platform for detecting resistant genotypes that can be integrated into agricultural system for crop improvement. On basis of scale proposed by Jo et al., ten genotypes including three resistant genotypes i.e. Chakwal3, Advanta5017, Chakwal4 and seven moderately resistant genotypes i.e., 32,351, Longi, 32,350, 32,354, Syngenta, Ghotki and Greenfire^36^(Figure. 1-b; Supplementary Table S5 online).

In current study the multivariate analysis or principal component analysis (PCA) helped to evaluate the significant genotypes for PcRR resistance using various traits and also showed the interrelation among these traits^37,38^. The Fig. 3-b depicted the PCA biplot based on PC1 and PC2 confirmed the significance of genotypes Chakwal3, Chakwal4, Advanta-5017 and Greenfire in the current study by finding them away from the central point. Therefore, these genotypes were also observed as major contributors^39^. According to Akand et al., the vectors in PCA biplot with acute angle show positive correlation, obtuse angle show negative correlation while right angle show zero correlation among trait^40^. Therefore, Fig. 3b-c confirmed the obtained findings by the existence of significantly positive association of STI with the yield per plant under both the control and the infected conditions. Moreover, the currently analyzed PCA showed that traits STI and CV are oppositely correlated with RCI, RLD, DI and DSI traits that ease the selection of compatible disease resistant genotypes from chilli germplasm^41,42^.

Numerous genes were discovered that encodes the regulation of disease resistance and susceptibility in plants by detecting pathogen invasion through signalling the plant components for sudden defense reaction^43,44^. The resistance mechanism in chilli plants is due to presence of R genes that are able to control 45 different *Phytophthora capsici* races^45^. Therefore, using conventional breeding methods for PcRR resistance in chilli is complicated and challenging that invokes the use of marker assisted selection for incorporating PcRR resistance in chilli^46^. However, marker identification followed by validation is critical prerequisite in the current study. Here, in the current study the previously identified 34 SSR markers were amplified using seventy-eight chilli genotypes and were validated to get trait specific significant markers^23^ (Table 2). Currently, the validation studies have obtained of 34 SSR markers that resulted in detection of five significant trait specific SSR markers. Therefore, trait specific markers including Hpms1172, CAMS177, CAMS066, CA06g27450 and CAMS173, whereas, Kumar, Kambham, Reddy, Sriram, & Singh reported one SSR marker (HpmsE034), Bongiorno et al., reported one SNP-based CAPS marker (*Phyto* CAPS), Zhang et al. reported seven KASP markers and Moreira et al. reported AFLP markers (Mcaa14/Eacg, Mcaa15/ Eacg) for PcRR resistance in chilli^18,47–49^. Interestingly, in the present study the both the SSR markers i.e. Hpms1172 and CAMS177 has shown significance for stress tolerance index, Moreover, the current finding will be helpful in enhancement of crop productivity, that is dependent on disease resistance in all crops^50^. In accordance with present results the study of Pawar et al. the SSR marker RM302 was found significant for days to 50% flowering as well as for days to maturity trait^51^. Moreover, the validation studies of Zeng, Meredith, Gutiérrez, & Boykin depicted that marker BNL285 was found associated with traits lint percentage and boll weight in cotton crop^52^. Moreover, the significant chilli genotypes that reside in these markers can be used in MAS for enhancement of yield as well as for disease resistance. The significant trait specific association of markers was also obtained in Pea and rice using the student’s *t* test^51,53^. The associated markers were considered as most reliable markers to utilize in crop improvement^54^. Therefore, the validation of trait specific markers is an important step to precede marker assisted selection^49^.

Moreover, current validation of markers confirmed the significance of commonly identified genotypes, Chakwal3, Greenfire, Advanta5017 and Chakwal4 for all five markers (Table 3; Supplementary Table S7 online; Figs. 4 and 5; Supplementary Figure. S1a-e online). Many commercial varieties are either partially resistant or even highly susceptible to *Phytophthora* root rot. Therefore, the identification of trait specific markers for PcRR resistance would be helpful for breeders to detect resistant genotypes in shortest time^55^. Previously, Kumar, Kambham, Reddy, Sriram, & Singh reported two, whereas, Wang, Wang, Guo, Yang, & Shen identified one genotype through marker assisted selection (MAS) for PcRR resistance in chilli^13,18^. The detected resistant genotypes were also found to be high yielding (Fig. 1-a; Supplementary Table S5 online). The accuracy of previously detected markers significantly shows variations if used for different chilli germplasms for disease related traits as observed in the study of Siddique et al., where 117 SNP marker associated with PcRR resistance were identified^56^. This variation in phenotype-genotype interaction is an important obstruction that limits the efficacy of MAS, making it challenging for PcRR resistance. The detected markers for PcRR resistance will lay the foundation for the molecular profiling of PcRR resistance in future breeding programmes. Therefore, there is dire need of continuous practice for detecting resistant genotypes through marker assisted selection (MAS) for PcRR resistance in chilli.

## Conclusion

*Phytophthora capsici* root rot (PcRR) is a destructive oomycete that difficult to control due to its polygenic nature. Therefore, the present study was planned for validation of previously reported markers to detect significant trait specific markers as well as marker assisted selection of PcRR resistant chilli genotypes. Furthermore, the validation of 34 previously detected SSR markers resulted in five significant trait specific markers. Furthermore, the association of SSR marker CAMS066 was observed with trait RCI, CA06g27450 with trait DI and CAMS173 with trait RLD. However, both the SSR markers Hpms1172 and CAMS177 were found associated with trait STI. The marker assisted selection confirmed the significance of phenotypically detected superior genotypes (Chakwal3, Greenfire, Advanta5017 and Chakwal4) by appearance of bright bands on gel pictures. The obtained marker assisted selection facilitates the early detection of high yielding resistant genotypes against drastic pathogen *Phytophthora capsici*. Therefore, the obtained significant SSR markers and marker assisted selection of chilli genotypes would be helpful in incorporating PcRR resistance in chilli hybrids for future breeding programs.

## Materials and methods

### Nursery preparation and experiment layout

The germplasm of 78 chilli genotypes was obtained from Plant Genetic Resource Centre (PGRI), National Agriculture Research Centre (NARC), Islamabad, Pakistan (Supplementary Table S1 online). The collected seeds were haloprimed by soaking chilli seeds in 3% KNO_3_ solution for 24 h to enhance germination ability of seeds^57^. Moreover, primed seeds were sown in trays filled with coco-peat in March 2020 (all experimental studies and experimental materials involved in this research are in full compliance with relevant institutional, national and international guidelines and legislation). Whereas, in May 2020, the two sets of chilli seedlings were transplanted at Plant Pathology section, Agriculture Research Institute Mingora, Swat, Pakistan (34.77 °N-72.34 °E). Each set consists of all 78 chilli genotypes (10 plants/genotype). One set of chilli seedlings was transplanted in field as the control condition, whereas second set was transplanted on PcRR infected sick bed as the infected condition. *Phytophthora capsici* root rot (PcRR) infected sick bed was prepared and maintained according to Shaw et al.^25^. Plants were transplanted with 60 cm of row to row distance and 45 cm of plant to plant distance in augmented block design (ABD) having four blocks. Nineteen chilli genotypes along with two checks were placed in each block. Cultural practices like irrigation, hoeing, roughing of weeds and application of NPK fertilizer etc. were applied as per recommendations. The crop was fertilized with N: P:K (20:20:20) solution in split doses. Furthermore, the screening and phenotyping was practiced on raised crop.

### Phenotyping

#### Disease scoring

When symptoms started appearing on plants, the scoring was observed on basis of disease scoring table (Supplementary Table S2 online) proposed by Bosland & Lindsey^58^ with few modifications to find out disease incidence (DI) % and disease severity index (DSI) % according to Aklilu, Ayana, Abebie, & Abdissa^59^,

$$Disease{\text{ }}incidence\left( {DI} \right)\% = {\mkern 1mu} {\mkern 1mu} \frac{{Number{\text{ }}of{\text{ }}infected{\text{ }}plants{\text{ }}per{\text{ }}row}}{{Total{\text{ }}number{\text{ }}of{\text{ }}plants{\text{ }}per{\text{ }}variety}} \times 100$$

$$Disease{\text{ }}severity{\text{ }}index\left( {DSI} \right)\% = \,{\mkern 1mu} \frac{{\left[ {\left( {0 \times a} \right) + \left( {1 \times b} \right) + \left( {2 \times c} \right) + \left( {3 \times d} \right) + \left( {4 \times e} \right) + \left( {5 \times f} \right)} \right]}}{{\left[ {\left( {a + b + c + d + e + f} \right) \times 5} \right]}} \times 100$$

where a, b, c, d, e and f refer to the numbers of plants fall under each score of scoring scale (0 to 5) related to their respective disease symptoms. The selection of genotypes was subjected to the model proposed by Jo et al. for classification of resistance level^36^. The genotype was considered as R: resistant if DI < 20%, MR: moderately resistant if DI = 20–50% and S: susceptible if DI > 50%.

#### Stress tolerance index (STI)

Yield per plant (YPP) of all the chilli genotypes was recorded for both the control as well as for the infected conditions. Moreover, on basis of yield data, stress tolerance index (STI) was calculated using formula proposed by Fernandez^60^,

$$Stress{\text{ }}tolerance{\text{ }}index\left( {STI} \right) = \left( {YP{P_c} \times YP{P_i}} \right)/YPP{C^2}$$

Where, YPP_c_ = yield of genotype under control condition, YPP_i_ = yield of genotype under infected condition and YPPC = mean yield of all genotype under control condition. A higher STI value for any genotype indicates its greater ability to tolerate disease and potentially achieve a higher yield.

#### Relative cell injury (%)

Relative cell injury (RCI) % was determined on the basis of electrolyte leakage following the modified method devised by Widmer, Graham, & Mitchell^61^. Initially, a set of ten root tips (15 mm) of each genotype from the control and the infected plants were collected. Each set of root tips were washed thrice in sterile, double-distilled water to remove any residual electrolytes and were placed in a test tube containing 15 ml of sterile, deionized distilled water. The samples were placed at room temperature for 24 h and the conductivity of the solution was measured using Electric Conductivity Meter (EC 215, Hanna Instruments). All test tubes were then sealed with cotton swabs and autoclaved for 15 min at 121 °C temperature to kill all cells. Samples were equilibrated to room temperature to take final conductivity.

The electrolyte leakage percentage was measured using following functions:

$$Electrolyte{\text{ }}Leakage{\left( {EL} \right)_{control}}\% = 1 - {C_1}/{C_2} \times 100,$$

$$Electrolyte{\text{ }}Leakage{\left( {EL} \right)_{infected}}\% = 1 - {T_1}/{T_2} \times 100,$$

Furthermore, relative cell injury (%) was calculated by using EL (%) value by following the formula proposed by Nijabat^62^.

$$Relative{\text{ }}cell{\text{ }}injury\left( {RCI} \right)\% = 1 - \left( {E{L_{infected}}\% /E{L_{control}}\% } \right) \times 100,$$

whereas, C_1_ = EL_control_% before autoclave, C_2_ = EL_control_% after autoclave, T_1_ = EL_infected_% before autoclave, T_2_ = EL_infected_% after autoclave.

#### Cell viability (%)

For measuring cell viability (CV) %, ten root tips from each observation per genotype (3 observations/genotype) were obtained from plants of the control condition as well as from the infected condition separately. The collected samples were washed with sterile, double-distilled water and were subjected to Evans blue aqueous solution (dye) with the volume of 0.25% (v/v) for 15 min, as per method proposed by Hameed, Keitel, Ahmad, Mahmood, & Trethowan^63^. The dyed root tips were then extensively washed separately with distilled water for 30 min to remove excess and unbound dye. The bounded dye to the dead cells was then solubilized in 50% (v/v) ethanol with 1% (w/v) SDS (Sodium Dodecyl Sulfate) at 60 °C for 30 min. The optical density (OD) was quantified by measuring absorbance at 600 nm from Spectrophotometer^64^. Another set of root tips from both the conditions was subjected to autoclave for killing cells, then treated with dye and latterly OD was measured. Cell viability was calculated using following formula.

$$Cell{\text{ }}viability\left( {CV} \right)\% = \frac{{Infected\left( {O{D_{autoclaved}} - O{D_{non - autoclved}}} \right)}}{{Control\left( {O{D_{autoclaved}} - O{D_{non - autoclved}}} \right)}} \times 100$$

#### Relative leaf damage (%)

The relative leaf damage (RLD) % was determined by detecting Hydrogen Peroxide (H_2_O_2_) by using DAB (3,3’-Diaminobenzidine) staining method as proposed by Daudi & O’Brien^65^. Young growing leaves (3 leaves/plant) of control/infected chilli plants were placed in 6 well micro titre plates, separately. Two ml of the Na_2_HPO_4_ DAB staining solution was poured in plates containing sample leaves. Moreover, solution from microtitre plates was discarded through vacuum infiltration, by placing plates in a dessicator for 5 min. Plates were then wrapped with aluminum foil and were then placed on a shaker for 4–5 h at 80–100 rpm speed. After incubation, foil was removed and solution was replaced by bleaching solution (ethanol: acetic acid: glycerol; 3:1:1). Chlorophyll on leaves was bleached by placing plates in a boiling water bath (90–95 °C) for 15 min. Formation of brown precipitates in plates having inoculated leaves was due to DAB reaction with the hydrogen peroxide. Few precipitates were found in plates with un-inoculated leaves. The stains on leaves were captured by placing treated leaves on white surface under uniform light. The total leaf area and DAB-stained leaf area of chilli leaves from both the control and the infected conditions was obtained by uploading photographs of DAB-stained leaves in Image J. software. The percent leaf area for both control and infected condition was calculated using formula proposed by Li et al.^66^.

$$Percent{\text{ }}leaf{\text{ }}are{a_{infected}} = DAB{\text{ }}stained{\text{ }}leaf{\text{ }}area/total{\text{ }}leaf{\text{ }}area \times 100$$

$$Percent{\text{ }}leaf{\text{ }}are{a_{control}} = DAB{\text{ }}stained\,leaf\,area/total\,\,leaf\,area \times 100$$

Furthermore, relative leaf damage % was calculated by using following formula:

$$Relative{\text{ }}leaf{\text{ }}damage\left( {RLD} \right)\% = Percent{\text{ }}leaf{\text{ }}are{a_{infected}}/Percent{\text{ }}leaf\,are{a_{control}} \times 100$$

#### Data analysis for phenotypic traits

Collected phenotypic data was statistically computed using R (version: 1.3.1093) computer software^67^. The agricolae R package was used for analysis of variance (ANOVA) according to d Steel & Torrie^68^, and least significant increase (LSI) was obtained according to Federer & Raghavarao^69^ for Augmented Block Design (ABD). LSI was added to the mean value of each check and thus resulted value of superior check was compared with the adjusted values of other genotypes. The genotype better from superior check (mean _check_ +LSI _check_) with maximum value was termed as best performing genotype among the studied germplasm. Furthermore, FactoMineR R package was used for determining principal component analysis (PCA) and constructing PCA-biplot with or without ellipses.

### Validation of markers and marker assisted selection (MAS)

#### DNA extraction and molecular profiling

Genomic DNA of 78 chilli genotypes was extracted according to the CTAB (cetyl trimethylammonium bromide) procedure proposed by Doyle^70^. The concentration and purity were assessed by observing absorbance ratio at 260:280 nm with a NanoDrop™ 1000 Spectrophotometer (Thermo Scientific, Germany) using 1 µL of each sample. The DNA of all 78 chilli genotypes were amplified using previously identified 34 polymorphic SSRs^23^ (Supplementary Table S3 online). PCR analysis was carried out in a reaction volume of 25 µL using method proposed by McGregor et al.^71^. The reaction mixture contained 1 ng template DNA, 0.2 U Taq polymerase, 0.4 µM of forward primer, 0.4 µM of reverse primer and 0.4 mM dNTPs. DNA amplification was carried out in the thermal cycler (T100, BIORAD, USA). PCR was set with 1-minute initial denaturation, followed by 35 cycles of denaturation at 95 °C for 25 s, annealing (specified for each primer for 25 s) and extension at 72 °C for 30 s. The reaction was completed by a final extension at 72 °C for 15 min. Samples were kept at hold at 4 °C after the final step. The amplified fragments were run along with 1 kb DNA ladder (Thermofisher), on 1.5% agarose gel and observed under Gel Documentation System Syngene (Model: InGenius3) and bands were recorded accordingly. The appearance of visually bright bands on gel pictures were considered as present, whereas considered as absent in case of absence of bands.

#### Statistical analysis for validation of markers

All markers were individually analyzed by using Student’s *t*-test^72^ as reported by Pawar, Suresh, Hittalmani, BC, & Biradar^51^.

$$S{p^2} = \frac{{{S_1}^2\left( {{n_1} - 1} \right) + {\text{ }}{S_2}^2\left( {{n_2} - 1} \right)}}{{{n_1} + {\text{ }}{n_2} - 2}}$$

$$Student'st - test = {\overline X _1} - {\overline X _2}/\surd \left( {S{p^2}\left[ {1/{n_1} + {\text{ }}1/{n_2}} \right]} \right)$$

${\overline X _1}$ = mean of trait that reside under genotypic value 1, ${\overline X _2}$ = mean of trait that reside under genotypic value 0, Sp^2^= pooled variance, n_1_ = number of genotypes in ${\overline X _2}$ and n_2_ = number of genotypes in ${\overline X _2}$. Moreover, the data was considered as significant on basis of threshold values i.e. *P*-value (*P* < 0.05).

#### Principal coordinate analysis

Furthermore, principal coordinate analysis (PCoA) was computed using GenAlex software^73^.

## Electronic supplementary material

Below is the link to the electronic supplementary material.

Supplementary Material 1

## Data Availability

All data generated or analyzed during this study are included in this published article (and its Supplementary Information files).

## Declarations

### Statement on guidelines

All experimental studies and experimental materials involved in this research are in full compliance with relevant institutional, national and international guidelines and legislation.

## References

[1] Granke, L. L., Quesada-Ocampo, L., Lamour, K. & Hausbeck, M. K. advances in research on Phytophthora capsici on vegetable crops in the United States. *Plant. Dis.* **96**, 1588–1600 (2012). 30727465 10.1094/PDIS-02-12-0211-FE

[2] Fao.org/faostat/en/#data/QCL. Crops and livestock products. *Food and Agriculture Organization of the United Nations, Rome, Italy.* (2021).

[3] Economic survey of Pakistan. Agriculture. Ministry of Finance, Government of Pakistan. *Islamabad Pakistan* 19–30 (2022–2023).

[4] Lamour, K. H., Stam, R., Jupe, J. & Huitema, E. The oomycete broad-host‐range pathogen Phytophthora Capsici. *Mol. Plant. Pathol.* **13**, 329–337 (2012). 22013895 10.1111/j.1364-3703.2011.00754.x PMC6638677

[5] Islam, S. Z., Babadoost, M., Lambert, K. N., Ndeme, A. & Fouly, H. M. Characterization of Phytophthora Capsici isolates from processing pumpkin in Illinois. *Plant. Dis.* **89**, 191–197 (2005). 30795223 10.1094/PD-89-0191

[6] Rai, G. S. & Guest, D. I. Drainage, animal manures and fungicides reduce Phytophthora wilt (caused by Phytophthora Capsici) of Chilli (Capsicum annuum L.) in Bhutan. *Australas Plant. Pathol.* **50**, 169–177 (2021).

[7] Waszczak, C., Carmody, M. & Kangasjärvi, J. Reactive oxygen species in plant signaling. *Annu. Rev. Plant. Biol.* **69**, 209–236 (2018). 29489394 10.1146/annurev-arplant-042817-040322

[8] Ishaq, L. et al. Abundance of arbuscular mycorrhizal fungi in the rhizosphere of healthy and declining citrus in East Nusa Tenggara, Indonesia. *Asian J. Agric. Biol.* **2023**(3), 2023011. 10.35495/ajab.2023.011 (2023).

[9] Fichman, Y. & Mittler, R. Rapid systemic signaling during abiotic and biotic stresses: is the ROS wave master of all trades? *Plant. J.* **102**, 887–896 (2020). 31943489 10.1111/tpj.14685

[10] Taratima, W., Kunpratum, N. & Maneerattanarungroj, P. Effect of salinity stress on physiological aspects of pumpkin (Cucurbita moschata Duchesne. ‘Laikaotok’) under hydroponic condition. *Asian J. Agric. Biol.* 2**023**(2), 202101050. 10.35495/ajab.2021.01.050 (2023).

[11] Wang, Y. et al. Production, signaling, and scavenging mechanisms of reactive oxygen species in fruit–pathogen interactions. *Int. J. Mol. Sci.* **20**, 2994 (2019). 31248143 10.3390/ijms20122994 PMC6627859

[12] Du, J. S. et al. The dissection of R genes and locus Pc5. 1 in Phytophthora capsici infection provides a novel view of disease resistance in peppers. *BMC Genom.* **22**, 1–16 (2021). 10.1186/s12864-021-07705-z PMC8139160 34016054

[13] Wang, P., Wang, L., Guo, J., Yang, W. & Shen, H. Molecular mapping of a gene conferring resistance to Phytophthora Capsici Leonian race 2 in pepper line PI201234 (Capsicum annuum L). *Mol. Breed.* **36**, 1–11 (2016).

[14] Rehrig, W. Z., Ashrafi, H., Hill, T., Prince, J. & Van Deynze, A. CaDMR1 cosegregates with QTL Pc5. 1 for resistance to Phytophthora capsici in pepper (Capsicum annuum). *Plant. Genome*. **7**, plantgenome2014–plantgenome2003 (2014).

[15] Kim, N., Kang, W. H., Lee, J. & Yeom, S. I. Development of clustered resistance gene analogs-based markers of resistance to Phytophthora capsici in chili pepper. *Biomed Res. Int.* 1–12 (2018). (2018). 10.1155/2019/1093186 PMC6335758 30719438

[16] Basit, M. A. et al. Qualitative and quantitative phytochemical analysis, antioxidant activity and antimicrobial potential of selected herbs Piper betle and Persicaria odorata leaf extracts. *Asian J. Agric. Biol.* **2023**(3), 2023038. 10.35495/ajab.2023.038 (2023).

[17] Fatemi, R. et al. Screening barley genotypes in terms of some quantitative and qualitative characteristics under normal and water deficit stress conditions. *Asian J. Agric. Biol.* **2023**(2), 2022071. 10.35495/ajab.2022.071 (2023).

[18] Kumar, M., Kambham, M. R., Reddy, D. C. L., Sriram, S. & Singh, T. H. Identification of molecular marker linked to resistance gene loci against Indian isolate of Phytophthora Capsici L. causing root rot in Chilli (Capsicum annuum L). *Australas Plant. Pathol.* **51**, 211–220 (2022).

[19] Arpaci, B. B. & Karataş, K. Comparison of Chili pepper breeding populations for agronomic traits and polygenic resistance to Phytophthora blight. *Hortic. Bras.* **38**, 12–20 (2020).

[20] Thabuis, A. et al. Phenotypic and molecular evaluation of a recurrent selection program for a polygenic resistance to Phytophthora capsici in pepper. *Theor. Appl. Genet.* **109**, 342–351 (2004). 15014880 10.1007/s00122-004-1633-9

[21] Swarup, S. et al. Genetic diversity is indispensable for plant breeding to improve crops. *Crop Sci.* **61**, 839–852 (2021).

[22] Alsaleh, A. et al. Marker-assisted selection and validation of DNA markers associated with cadmium content in durum wheat germplasm. *Crop Pasture Sci.* **73** (8) (2022).

[23] Bukhari, T., Rana, R. M., Hassan, M. U., Naz, F. & Sajjad, M. Genetic diversity and marker trait association for phytophthora resistance in Chilli. *Mol. Biol. Rep.* **49**, 5717–5728 (2022). 35701684 10.1007/s11033-022-07635-3

[24] Moreira-Morrillo, A. A., Monteros-Altamirano, Á., Reis, A. & Garcés-Fiallos, F. R. Phytophthora capsici on Capsicum Plants: A Destructive Pathogen in Chili and Pepper Crops. (2022).

[25] Iribarren, M. J., Steciow, M., González, B. & Nardelli, M. Prevalence and aetiology of Phytophthora fruit and stem rot of solanaceous and cucurbitaceous crops in the Pampas region of Argentina. *J. Plant. Pathol.* **101**, 481–489 (2019).

[26] Shaw, R. K. et al. Establishing a high throughput screening method for large scale phenotyping of castor genotypes for resistance to Fusarium wilt disease. *Phytoparasitica*. **44**, 539–548 (2016).

[27] Mansour, E. et al. Multidimensional evaluation for detecting salt tolerance of bread wheat genotypes under actual saline field growing conditions. *Plants*. **9**, 1324 (2020). 33036311 10.3390/plants9101324 PMC7601346

[28] Rahman, S. et al. (ed, U.) Influence of Tryptophan on the growth, yield and quality of Chilli with and without fertilizer. *Pure Appl. Biology* **10** 4 1287–1302 (2021).

[29] Sanogo, S. & Ji, P. Integrated management of Phytophthora capsici on solanaceous and cucurbitaceous crops: current status, gaps in knowledge and research needs. *Can. J. Plant. Pathol.* **34**, 479–492 (2012).

[30] Aktar-Uz-Zaman, M. et al. Selection of Lentil (Lens Culinaris (Medik.)) Genotypes Suitable for High-Temperature Conditions Based on Stress Tolerance Indices and Principal Component Analysis. *Life* 12, 1719 (2022). 10.3390/life12111719 PMC9698439 36362874

[31] Kumar, R., Mina, U., Gogoi, R., Bhatia, A. & Harit, R. C. Effect of elevated temperature and carbon dioxide levels on maydis leaf blight disease tolerance attributes in maize. *Agric. Ecosyst. Environ.* **231**, 98–104 (2016).

[32] Kalve, S. & Gali, K. K. Tar’an, B. Genome-wide association analysis of stress tolerance indices in an interspecific population of chickpea. *Front. Plant. Sci.* **13**, 933277 (2022). 36061786 10.3389/fpls.2022.933277 PMC9437449

[33] Ahmadi, J., Pour-Aboughadareh, A., Fabriki-Ourang, S., Mehrabi, A. A. & Siddique, K. H. M. Screening wheat germplasm for seedling root architectural traits under contrasting water regimes: potential sources of variability for drought adaptation. *Arch. Agron. Soil. Sci.* **64**, 1351–1365 (2018).

[34] Mudi, N., Mahapatra, S. & Das, S. Assessment of Helminthosporium blight resistance in barley using disease stress tolerance index. *Indian Phytopathol.* **69**, 24–31 (2016).

[35] Sharf, W., Javaid, A., Shoaib, A. & Khan, I. H. Induction of resistance in Chili against Sclerotium rolfsii by plant-growth-promoting rhizobacteria and Anagallis arvensis. *Egypt. J. Biol. Pest Control*. **31**, 1–11 (2021).

[36] Jo, S. J. et al. Resistance of Chili pepper cultivars to isolates of Phytophthora capsici. *Hortic. Sci. Technol.* **32**, 66–76 (2014).

[37] Mubushar, M. et al. Assessing the Suitability of Multivariate Analysis for Stress Tolerance Indices, Biomass, and Grain Yield for Detecting Salt Tolerance in Advanced Spring Wheat lines irrigated with saline water under Field conditions. *Agronomy*. **12**, 3084 (2022).

[38] Singh, P., Jain, P. K. & Tiwari, A. Principal component analysis approach for yield attributing traits in Chilli (Capsicum annum L.) genotypes. *Chem. Sci. Rev. Lett.* **9**, 87–91 (2020).

[39] Kaiser, H. F. An index of factorial simplicity. *Psychometrika*. **39**, 31–36 (1974).

[40] Akand, M. et al. Parent selection for Intercrossing in Chili (Capsicum annuum L.) through Multivariate Genetic Divergence Analysis. *Mol. Plant. Breed.* **7**, (2016).

[41] Karipçin, Z., Seyidoğlu, G. & Mikail, N. Characterization of phytophthora capsici leonian resistance in some pepper genotypes by principal component analysis. (2018).

[42] Memon, A., Ahmad, R., Depar, M. S., Pathan, A. K. & Ibrar, D. Estimation of genetic divergence in Chilli pepper (Capsicum annuum L.) genotypes for morphological and fruit traits under hot climate of Umerkot, Sindh. *Pakistan J. Agric. Agric. Eng. Vet. Sci.* **37**, 21–28 (2021).

[43] Antolín-Llovera, M., Ried, M. K., Binder, A. & Parniske, M. Receptor kinase signaling pathways in plant-microbe interactions. *Annu. Rev. Phytopathol.* **50**, 451–473 (2012). 22920561 10.1146/annurev-phyto-081211-173002

[44] Wang, Y., Li, X., Fan, B., Zhu, C. & Chen, Z. Regulation and function of defense-related callose deposition in plants. *Int. J. Mol. Sci.* **22**, 2393 (2021). 33673633 10.3390/ijms22052393 PMC7957820

[45] Barchenger, D. W., Lamour, K. H. & Bosland, P. W. Challenges and strategies for breeding resistance in Capsicum annuum to the multifarious pathogen, Phytophthora capsici. *Front. Plant. Sci.* **9**, 628 (2018). 29868083 10.3389/fpls.2018.00628 PMC5962783

[46] Lee, J., Lee, W. P., Kang, B. C. & Yoon, J. B. Inheritance of resistance to Phytophthora root rot in Chili pepper depending on inoculum density and parental genotypes. *Korean J. Breed. Sci.* **44**, 503–509 (2012).

[47] Bongiorno, G. et al. Development and application of a cleaved amplified polymorphic sequence marker (Phyto) linked to the Pc5. 1 locus conferring resistance to Phytophthora Capsici in Pepper (Capsicum annuum L). *Plants*. **12**, 2757 (2023). 37570909 10.3390/plants12152757 PMC10421461

[48] Zhang, Z. et al. Development and validation of KASP markers for resistance to Phytophthora capsici in Capsicum annuum L. *Mol. Breed.* **43**, 20 (2023). 37313294 10.1007/s11032-023-01367-3 PMC10248700

[49] Moreira, A. F. P. et al. Genetic diversity, population structure and genetic parameters of fruit traits in Capsicum chinense. *Sci. Hortic. (Amsterdam)*. **236**, 1–9 (2018).

[50] Acharya, B., Dutta, S., Dutta, S. & Chattopadhyay, A. Breeding tomato for simultaneous improvement of processing quality, fruit yield, and dual disease tolerance. *Int. J. Veg. Sci.* **24**, 407–423 (2018).

[51] Pawar, P., Suresh, C. K., Hittalmani, S., BC, K. M. & Biradar, H. DNA marker-assisted analysis of recombinant inbred lines using trait-specific markers and candidate genes in Rice (Oryza sativa L). *Genes Genomes Genomics*. **6**, 48–51 (2012).

[52] Zeng, L., Meredith, W. R., Gutiérrez, O. A. & Boykin, D. L. Identification of associations between SSR markers and fiber traits in an exotic germplasm derived from multiple crosses among Gossypium tetraploid species. *Theor. Appl. Genet.* **119**, 93–103 (2009). 19360391 10.1007/s00122-009-1020-7

[53] Gawłowska, M. et al. Validation of molecular markers significant for flowering time, plant lodging, stem geometry properties, and raffinose family oligosaccharides in pea (Pisum sativum l). *Agriculture*. **12**, 1125 (2022).

[54] Diapari, M., Sindhu, A., Warkentin, T. D. & Bett, K. Tar’an, B. Population structure and marker-trait association studies of iron, zinc and selenium concentrations in seed of field pea (Pisum sativum L). *Mol. Breed.* **35**, 1–14 (2015).

[55] Quesada-Ocampo, L. M. et al. Phytophthora capsici: recent progress on Fundamental Biology and Disease Management 100 years after its description. *Annu. Rev. Phytopathol.* **61**, (2023). 10.1146/annurev-phyto-021622-103801 37257056

[56] Siddique, M. I. et al. Identifying candidate genes for Phytophthora capsici resistance in pepper (Capsicum annuum) via genotyping-by-sequencing-based QTL mapping and genome-wide association study. *Sci. Rep.* **9**, 9962 (2019). 31292472 10.1038/s41598-019-46342-1 PMC6620314

[57] Maiti, R., Rajkumar, D., Jagan, M., Pramanik, K. & Vidyasagar, P. Effect of seed priming on seedling vigour and yield of tomato and Chilli. *Int. J. Bio-resource Stress Manag*. **4**, 119–125 (2013).

[58] Bosland, P. W. & Lindsey, D. L. *A Seedling Screen for Phytophthora Root Rot of Pepper, Capsicum Annuum*. (1991).

[59] Aklilu, S., Ayana, G., Abebie, B. & Abdissa, T. Screening for resistance sources in local and exotic hot pepper genotypes to Fusarium wilt (Fusarium oxysporium) and associated quality traits in Ethiopia. *Adv. Crop Sci. Technol.* **6**, 367–376 (2018).

[60] Fernandez, G. C. J. Effective selection criteria for assessing plant stress tolerance. in *Proceeding of the International Symposium on Adaptation of Vegetables and other Food Crops in Temperature and Water Stress, Aug. 13–16, Shanhua, Taiwan, 1992* 257–270 (1992).

[61] Widmer, T. L., Graham, J. H. & Mitchell, D. J. Histological comparison of fibrous root infection of disease-tolerant and susceptible citrus hosts by Phytophthora nicotianae and P. Palmivora. *Phytopathology*. **88**, 389–395 (1998). 18944916 10.1094/PHYTO.1998.88.5.389

[62] Nijabat, A. et al. Cell membrane stability and relative cell injury in response to heat stress during early and late seedling stages of diverse carrot (Daucus carota L.) germplasm. *Hortscience*. **55**, 1446–1452 (2020).

[63] Hameed, M., Keitel, C., Ahmad, N., Mahmood, T. & Trethowan, R. Screening of tomatoes germplasm for heat stress tolerance under controlled conditions. *Procedia Environ. Sci.* **29**, 173–174 (2015).

[64] Gracia-Medrano, R. M. E. & de Lourdes Miranda-Ham, M. Analysis of elicitor-induced cell viability changes in Lycopersicon esculentum Mill. suspension culture by different methods. *Vitr. Cell. Dev. Biol.* 39, 236–239 (2003).

[65] Daudi, A. & O’Brien, J. A. Detection of hydrogen peroxide by DAB staining in Arabidopsis leaves. *Bio-protocol*. **2**, e263–e263 (2012). 27390754 PMC4932902

[66] Li, Y. et al. Streptomyces pactum Act12 controls tomato yellow leaf curl virus disease and alters rhizosphere microbial communities. *Biol. Fertil. Soils*. **55**, 149–169 (2019).

[67] Core, R. T. R: A language and environment for statistical computing (2019). https://cir.nii.ac.jp/crid/1370579814635375110

[68] d Steel, R. G. & Torrie, J. H. *Principles and Procedures of Statistics: A Biometrical Approach* (McGraw-Hill, 1986).

[69] Federer, W. T. & Raghavarao, D. On augmented designs. *Biometrics* 29–35 (1975).

[70] Doyle, J. J. Isolation of plant DNA from fresh tissue. *Focus (Madison)*. **12**, 13–15 (1990).

[71] McGregor, C. et al. Genotypic and phenotypic variation among pepper accessions resistant to Phytophthora capsici. *HortScience*. **46**, 1235–1240 (2011).

[72] Gösset, W. S. The probable error of a mean. *Biometrika*. **6**, 1–25 (1908).

[73] Peakall, R. & Smouse, P. E. GenAlEx 6.5: genetic analysis in Excel. Population genetic software for teaching and research–an update. *Bioinformatics*. **28**, 2537e2539 (2012). 22820204 10.1093/bioinformatics/bts460 PMC3463245

