# Supplementary material for: Validation of SSR markers for identification of high-yielding and Phytophthora Capsici root rot resistant chilli genotypes
Source: Sci Rep. 2024 Nov 19;14:28569. doi: 10.1038/s41598-024-79718-z (PMC11576959; doi:10.1038/s41598-024-79718-z)
Supplement: Supplementary file 1 — Supplementary Material 1 [file 41598_2024_79718_MOESM1_ESM.docx]

**List of tables**

Supplementary Table S1: Names of 78 chilli genotypes used in present study.

Supplementary Table S2: Disease scoring scale for screening of chilli genotypes against *Phytophthora* root rot.

Supplementary Table S3: List of 150 SSR markers along with primer sequences and reference.

Supplementary Table S4. Least significant increase (LSI) based mean performance of checks and adjusted means for phenotypic traits of chilli.

Supplementary Table S5: Disease incidence and classification of resistance level of 78 chilli genotypes against *Phytophthora capsici* root rot.

Supplementary Table S6: Principal component analysis (PCA) for phenotypic traits using 78 chilli genotypes.

Supplementary Table S7: Principal co-ordinate analysis (PC0A) of 78 chilli genotypes using 34 SSR markers.

Supplementary Table S1: Names of 78 chilli genotypes used in present study.

| **S.no** | **Genotype** | **S.no** | **Genotype** | **S.no** | **Genotype** | **S.no** | **Genotype** |
| --- | --- | --- | --- | --- | --- | --- | --- |
| 1 | 1787 | 21 | 24634 | 41 | 16/5 | 61 | Ghotki |
| 2 | 1791 | 22 | 32319 | 42 | 16/7 | 62 | Longi |
| 3 | 1792 | 23 | 32320 | 43 | 16/8 | 63 | PusaJawala |
| 4 | 1799 | 24 | 32321 | 44 | 16/9 | 64 | Syngenta |
| 5 | 16162 | 25 | 32324 | 45 | Skyway | 65 | Cdk-101 |
| 6 | 16163 | 26 | 32328 | 46 | Skyline2 | 66 | Advanta-5017 |
| 7 | 16168 | 27 | 32332 | 47 | 15/4 | 67 | Marvi558 |
| 8 | 16169 | 28 | 32333 | 48 | 15/5 | 68 | Greenstar |
| 9 | 20366 | 29 | 32335 | 49 | 15/6 | 69 | Greengold |
| 10 | 20372 | 30 | 32336 | 50 | KHHP-081A | 70 | Supersky-AB |
| 11 | 20451 | 31 | 32344 | 51 | 408 | 71 | Kalae542 |
| 12 | 20523 | 32 | 32350 | 52 | 1108 | 72 | HP-1410 |
| 13 | 20524 | 33 | 32351 | 53 | 1209 | 73 | Advanta-512 |
| 14 | 24621 | 34 | 32354 | 54 | 9905 | 74 | PH-264 |
| 15 | 24623 | 35 | 32362 | 55 | 59328 | 75 | Greenfire |
| 16 | 24624 | 36 | 32370 | 56 | Chakwal1 | 76 | HP1449 |
| 17 | 24625 | 37 | 32385 | 57 | Chakwal2 | 77 | 1031 |
| 18 | 24626 | 38 | 32390 | 58 | Chakwal3 | 78 | Disney |
| 19 | 24627 | 39 | 14/9 | 59 | Chakwal4 |  |  |
| 20 | 24629 | 40 | 16/4 | 60 | Chakwal5 |  |  |

Supplementary Table S2: Disease scoring scale for screening of chilli genotypes against *Phytophthora* root rot.

| **Disease score** | **Description** |
| --- | --- |
| 0 | No response, vigorous, healthy. |
| 1 | Slight root darkening, vigorous and healthy. |
| 2 | Brown roots, slight stunting, very small lesions on stems, lower leaves wilted and stunted plants. |
| 3 | Brown roots, small lesions on stems, lower leaves wilted and stunted plants. |
| 4 | Brown roots, large lesions on stems, girdling, and whole plant wilted and stunted. |
| 5 | Death |

Supplementary Table S3: List of 150 SSR markers along with primer sequences and reference.

| **SSRs** | **Forward Primer(5ʹ-3ʹ)** | **Reverse Primer(3ʹ-5ʹ )** | **References** |
| --- | --- | --- | --- |
| CA02g13320 | CCAAACGACCCCTTAGTGTG | GGCAAACAACTTATGTGGAA | (Meng et al., 2017) |
| CA01g19200 | TCCTCGTCATACCAAAAGCC | CCAAGCAACAAGAGAAAGGG | (Meng et al., 2017) |
| CA06g27450 | ATTCATGTTCGTTTGTGCGA | CCTACCCATAGACAGCAGCC | (Meng et al., 2017) |
| Hpms168 | GCCCCGATCAATGAATTTCAAC | TGATTTTTGGGTGGAGAGAAAACC | (Lee, Nahm, Kim, & Kim, 2004) |
| Hpms1172 | GGGTTTGCATGATCTAAGCATTTT | CGCTGGAATGCATTGTCAAAGA | (Lee, Nahm, Kim, & Kim, 2004) |
| Hpms16 | TCCATAACTTCACCCATGAGTATGA | GCAACACCCACATTCCCTTCTC | (Lee, Nahm, Kim, & Kim, 2004) |
| Hpms162 | CATGAGGTCTCGCATGATTTCAC | GGAGAAGGACCATGTACTGCAGAG | (Lee, Nahm, Kim, & Kim, 2004) |
| Hpms1148 | GGCGGAGAAGAACTAGACGATTAGC | CCACCCAATCCACATAGACG | (Lee, Nahm, Kim, & Kim, 2004) |
| Hpms11 | TCAACCCAATATTAAGGTCACTTCC | CCAGGCGGGGATTGTAGATG | (Lee, Nahm, Kim, & Kim, 2004) |
| CAMS173 | CAACCGCCAGTAGACAGGTT | GTGCGTGTGCGTGTGTGTAT | (Minamiyama, Tsuro, & Hirai, 2006) |
| CAMS227 | TTTGTCCTTTAATTCACCTTTTGA | GCATCAAAATAAGGATAAAGTTATGG | (Minamiyama, Tsuro, & Hirai, 2006) |
| CAMS396 | GTCGGCCGTCATTCACTATT | AGCTTGATGCACCTGGTCTT | (Minamiyama, Tsuro, & Hirai, 2006) |
| CAMS177 | ATTCTCTACCCCTGCCTGTG | CTCAGGAGATGTCCCACGAT | (Minamiyama, Tsuro, & Hirai, 2006) |
| CAeMS138 | ACACACACAATTTCCCTCACTCAC | GTTTCTCTCAAATCCCTCCGTTGTTC | (Minamiyama, Tsuro, & Hirai, 2006) |
| CAMS095 | CGCTAGCATGACACTCAAGG | AAACGGCAAGGCTACACATC | (Minamiyama, Tsuro, & Hirai, 2006) |
| CAMS015 | TCATGTTGATTATGCTTTTGTTCA | CCATGTATTGTATGATACCTGAGAAA | (Minamiyama, Tsuro, & Hirai, 2006) |
| CAMS070 | CCCTGAACTTGTCCTCCAAA | GGGTATGGGGTGTAGGTGTG | (Minamiyama, Tsuro, & Hirai, 2006) |
| CAeMS144 | ATAACTTTGATTCCTAGTTCGGCG | GTTTGAACCCCCAATCATCATATCCTCA | (Minamiyama, Tsuro, & Hirai, 2006) |
| CAMS066 | AAAAACATGCACCAGTCCTT | CAACCGCCTGAATTTTCTCT | (Minamiyama, Tsuro, & Hirai, 2006) |
| CAMS871 | ACAAAGCATCGGCTGAAAAT | GCGACCAAGTACCAACAGGT | (Minamiyama, Tsuro, & Hirai, 2006) |
| CAMS194 | TCATGGAAAATTAACAACGCATA | GGGGGTTGGAGAAGAAAGTT | (Minamiyama, Tsuro, & Hirai, 2006) |
| CAMS644 | CGCATGAAGCAAATGTACCA | ACCTGCAGTTTGTTGTTGGA | (Minamiyama, Tsuro, &Hirai, 2006) |
| CAeMS073 | ATGCTTCTAAGAAACCCCACAACA | GTTTCTCATAAAGGGGTTGGGATTGA | (Minamiyama, Tsuro, & Hirai, 2006) |
| CAeMS015 | ATGCCTTGGTGGTGGTTAAATCTG | GTTTAGCGGTATGGACTGCGTACATCTT | (Minamiyama, Tsuro, & Hirai, 2006) |
| CAMS0632 | AACTCAGGTACACGGGATAAAA | TGTTTGCCACTGTATGTGTCTG | (Minamiyama, Tsuro, & Hirai, 2006) |
| EPMS418 | ATCTTCTTCTCATTTCTCCCTTC | TGCTCAGCATTAACGACGTC | (Nagy, Stagel, Sasvari, Röder, & Ganal, 2007) |
| EPMS417 | CGCATATACATACATAAATTCTTTC | TCAACATCTCACCGAAGCTG | (Nagy, Stagel, Sasvari, Röder, & Ganal, 2007) |
| EPMS404 | TCTCTCTCTACATCTCTCCGTTG | TGTCGTTCGTCGACGTACTC | (Nagy, Stagel, Sasvari, Röder, & Ganal, 2007) |
| GPMS159 | GGAAGATCCCTTGAATGAGTATGTCTC | GGCTGAAAATGTCTGATGGAACTGG | SOL CAP |
| T0633 | GATGGGCTATGCTTGCTGTT | ACATCCCCAATGTTGTTGTG | SOL CAP |
| GP20087 | CCCTCTCCTCAATTCACA | CCTTTACCCCTAAATTTGAT | SOL CAP |
| CA516044 | ATCTTCTTCTCATTTCTCCCTTC | TGCTCAGCATTAACGACGTC | SOL CAP |
| GU295217 | TTTCGGATTGCCCTATGCTTGTT | AAATTTGTGAGGGCTGTTAGGT | NCBI |
| EF100893 | GTTTGGTCTTGTGGGGTCAC | GGCTTTTCTCCACCATTCAC | NCBI |
| SOLCAP=Solanacea Agriculture Project; NCBI=National Center for Biotechnology Information. | | | |

Supplementary Table S4. Least significant increase (LSI) based mean performance of checks and adjusted means for phenotypic traits of chilli.

| **Genotype** | **Block** | **YPPc** | **YPPi** | **STI** | **RCI** | **CV** | **DI** | **DSI** | **RLD** |
| --- | --- | --- | --- | --- | --- | --- | --- | --- | --- |
| LSI(0.05) |  | 19.34 | 10.24 | 0.14 | 3.41 | 0.42 | 0.36 | 0.91 | 0.89 |
| Chakwal3(Mean+LSI) |  | 99.01 | 100.93 | 12.12 | 23.16 | 93.31 | 7.17 | 2.01 | 17.18 |
| Chakwal4(Mean+LSI) |  | 68.33 | 67.77 | 7.94 | 31.79 | 78.43 | 19.22 | 2.34 | 0.90 |
| 1787 | I | 7.04 | 17.53 | 0.36 | 69.17 | 51.19 | 98.76 | 37.11 | 1.23 |
| 1791 | I | 6.69 | 3.74 | 0.16 | 79.17 | 77.75 | 99.65 | 59.49 | 10.13 |
| 1792 | I | 3.09 | 14.51 | 0.22 | 61.25 | 29.14 | 79.77 | 23.83 | 1.45 |
| 1799 | I | 4.77 | 6.50 | 0.12 | 77.14 | 50.51 | 56.32 | 4.67 | 2.89 |
| 16162 | I | 1.64 | 9.74 | 0.02 | 72.22 | 83.61 | 59.98 | 3.67 | 13.84 |
| 16163 | I | 7.57 | 3.98 | 0.17 | 57 | 17.79 | 76.32 | 5.06 | 1.88 |
| 16168 | I | 23.73 | 35.19 | 2.25 | 81.25 | 78.12 | 59.88 | 5.00 | 1.39 |
| 16169 | I | 19.28 | 30.68 | 1.72 | 74.81 | 27.61 | 52.99 | 4.84 | 10.87 |
| 20366 | I | 5.54 | 5.23 | 0.14 | 77.11 | 22.78 | 99.10 | 67.17 | 16.26 |
| 20372 | I | 22.09 | 33.59 | 2.15 | 74.9 | 51.94 | 99.65 | 100 | 4.23 |
| 20451 | I | 2.61 | 8.85 | 0.05 | 72.56 | 85.36 | 55.78 | 3.83 | 10.24 |
| 20523 | I | 19.31 | 29.58 | 1.7 | 80.83 | 44.35 | 98.43 | 72.5 | 17.87 |
| 20524 | I | 4.72 | 6.69 | 0.12 | 88.06 | 54.99 | 99.65 | 74.27 | 0.96 |
| 24621 | I | 6.61 | 4.09 | 0.16 | 81.15 | 46.07 | 99.65 | 80.5 | 9.44 |
| 24623 | I | 8.91 | 20.42 | 0.61 | 35.63 | 55.56 | 99.65 | 100 | 38.10 |
| 24624 | I | 1.08 | 11.29 | 0.08 | 72.36 | 67.10 | 98.82 | 82.77 | 11.16 |
| 24625 | I | 7.54 | 3.98 | 0.17 | 46.25 | 29.05 | 86.56 | 29.21 | 0.67 |
| 24626 | I | 3.67 | 7.76 | 0.09 | 56.8 | 46.14 | 99.65 | 100 | 16.09 |
| 24627 | I | 0.34 | 11.03 | 0.04 | 39.29 | 18.29 | 97.81 | 16.5 | 16.41 |
| 24629 | II | 18.49 | 29.25 | 1.99 | 48.33 | 36.06 | 100 | 100 | 23.10 |
| 24634 | II | 1.50 | 9.24 | 0.35 | 42.74 | 48.11 | 100 | 50.34 | 88.92 |
| 32319 | II | 2.67 | 29.25 | 0.54 | 74.23 | 68.32 | 87.72 | 59.5 | 36.45 |
| 32320 | II | 8.59 | 9.24 | 0.92 | 75 | 35.21 | 99.89 | 70.34 | 3.63 |
| 32321 | II | 40.92 | 13.19 | 5.55 | 39.96 | 26.12 | 98.96 | 48.34 | 12.59 |
| 32324 | II | 5.63 | 19.19 | 0.23 | 52.81 | 46.41 | 83.62 | 26.99 | 3.34 |
| 32328 | II | 1.65 | 51.63 | 0.34 | 87.65 | 77.68 | 99.84 | 53.67 | 5.68 |
| 32332 | II | 32.85 | 5.19 | 3.39 | 72.05 | 79.86 | 80.28 | 8.39 | 4.07 |
| 32333 | II | 1.98 | 9.08 | 0.33 | 78.17 | 27.26 | 83.62 | 12.17 | 16.24 |
| 32335 | II | 14.94 | 43.55 | 1.45 | 56.4 | 28.59 | 77.05 | 6.67 | 2.34 |
| 32336 | II | 2.40 | 8.62 | 0.53 | 60.5 | 18.76 | 86.95 | 67.66 | 28.23 |
| 32344 | II | 3.85 | 25.80 | 0.27 | 53.5 | 16.05 | 100 | 52.67 | 1.90 |
| 32350 | II | 12.85 | 13.10 | 1.24 | 88.56 | 20.09 | 33.62 | 2.84 | 3.32 |
| 32351 | II | 1.54 | 6.98 | 0.49 | 53.55 | 59.66 | 20.50 | 1.45 | 8.68 |
| 32354 | II | 7.00 | 23.48 | 0.21 | 78.81 | 55.33 | 43.72 | 2.34 | 0.57 |
| 32362 | II | 5.28 | 12.36 | 0.23 | 60.73 | 46.59 | 99.22 | 64.67 | 38.68 |
| 32370 | II | 8.49 | 3.79 | 0.94 | 48.94 | 81.02 | 99.78 | 47.61 | 1.33 |
| 32385 | II | 0.94 | 5.18 | 0.44 | 70.04 | 30.10 | 100 | 55.94 | 25.79 |
| 32390 | II | 11.19 | 19.35 | 1.11 | 54.51 | 47.53 | 100 | 100 | 35.05 |
| 14/9 | III | 47.10 | 11.15 | 0.67 | 75.66 | 20.91 | 99.64 | 67.17 | 29.55 |
| 16/4 | III | 75.14 | 46.60 | 4.6 | 82.1 | 36.26 | 99.97 | 64.66 | 2.66 |
| 16/5 | III | 44.42 | 15.80 | 0.49 | 80.86 | 78.43 | 98.58 | 36.77 | 1.71 |
| 16/7 | III | 50.70 | 22.10 | 0.99 | 81.85 | 16.35 | 99.97 | 100 | 20.35 |
| 16/8 | III | 32.75 | 17.99 | 0.06 | 74.49 | 18.54 | 91.70 | 38.67 | 26.29 |
| 16/9 | III | 41.15 | 46.60 | 0.35 | 59.88 | 53.65 | 99.97 | 52.72 | 1.00 |
| Skyway | III | 35.83 | 15.80 | 0.13 | 50.22 | 74.05 | 79.97 | 31 | 11.80 |
| Skyline2 | III | 32.39 | 22.10 | 0.06 | 60.49 | 30.79 | 99.97 | 100 | 21.76 |
| 15/4 | III | 31.87 | 4.21 | 0.05 | 79.61 | 34.94 | 99.69 | 67.33 | 7.95 |
| 15/5 | III | 56.27 | 12.64 | 1.54 | 55.89 | 17.19 | 99.97 | 100 | 17.58 |
| 15/6 | III | 60.94 | 7.27 | 1.9 | 81.66 | 67.43 | 99.87 | 49.34 | 3.58 |
| KHHP081A | III | 47.49 | 3.86 | 0.72 | 35.06 | 38.18 | 76.64 | 10.5 | 4.14 |
| 408 | III | 41.70 | 3.02 | 0.36 | 76.81 | 44.86 | 99.97 | 63.84 | 10.13 |
| 1108 | III | 45.34 | 27.67 | 0.5 | 68.75 | 16.42 | 99.97 | 100 | 31.66 |
| 1209 | III | 37.02 | 29.14 | 0.16 | 65.17 | 67.48 | 99.41 | 69.67 | 21.24 |
| 9905 | III | 63.47 | 18.83 | 2.63 | 71.09 | 61.84 | 83.31 | 8.17 | 1.32 |
| 59328 | III | 61.44 | 12.54 | 2.22 | 68.75 | 42.75 | 99.30 | 89 | 2.56 |
| Chakwal1 | III | 40.19 | 16.72 | 0.3 | 73.36 | 77.51 | 83.31 | 13.5 | 3.07 |
| Chakwal2 | III | 46.10 | 7.90 | 0.63 | 71.42 | 25.77 | 49.97 | 4.16 | 9.24 |
| Chakwal5 | IV | 1.82 | 29.65 | 0.12 | 74.29 | 58.30 | 90.09 | 12.84 | 2.04 |
| Ghotki | IV | 9.48 | 27.47 | 0.77 | 43.41 | 48.63 | 47.08 | 6.67 | 2.70 |
| Longi | IV | 13.36 | 6.24 | 1.08 | 41.96 | 31.73 | 23.43 | 8.67 | 0.74 |
| PusaJawala | IV | 16.73 | 12.30 | 1.56 | 69.05 | 75.42 | 100 | 50.84 | 1.69 |
| Syngenta | IV | 0.91 | 5.07 | 0.15 | 82.57 | 19.58 | 46.86 | 5.5 | 1.62 |
| Cdk-101 | IV | 2.13 | 8.05 | 0.31 | 81.81 | 39.93 | 83.43 | 26 | 4.07 |
| Advanta5017 | IV | 59.93 | 4.24 | 9.89 | 39.73 | 43.04 | 14.71 | 1.34 | 0.57 |
| Marvi558 | IV | 51.05 | 15.50 | 5.83 | 59.38 | 47.90 | 100 | 56.5 | 44.66 |
| Greenstar | IV | 15.23 | 19.37 | 1.34 | 64.1 | 69.47 | 99.75 | 64.5 | 36.20 |
| Greengold | IV | 65.56 | 22.62 | 2.09 | 78.44 | 76.77 | 99.15 | 50.67 | 30.98 |
| SuperskyAB | IV | 29.52 | 5.07 | 2.22 | 85.83 | 85.46 | 79.20 | 10.66 | 48.76 |
| Kalae542 | IV | 7.52 | 8.05 | 0.04 | 68.16 | 73.26 | 98.80 | 51.34 | 23.31 |
| HP-1410 | IV | 55.81 | 54.94 | 6.62 | 70.87 | 57.92 | 99.59 | 64.34 | 31.31 |
| Advanta512 | IV | 33.10 | 57.02 | 3.4 | 85.89 | 85.15 | 100 | 31 | 18.76 |
| PH-264 | IV | 46.46 | 21.22 | 3.8 | 77.72 | 56.68 | 100 | 86.67 | 25.20 |
| Greenfire | IV | 56.71 | 58.97 | 10.09 | 28.11 | 94.17 | 46.76 | 3.17 | 14.65 |
| HP1449 | IV | 44.91 | 24.98 | 5.1 | 77.38 | 44.93 | 100 | 100 | 26.66 |
| 1031 | IV | 3.40 | 1.85 | 0.05 | 85.39 | 65.34 | 100 | 100 | 42.20 |
| Disney | IV | 3.03 | 45.07 | 0.33 | 77.1 | 29.65 | 98.70 | 30.34 | 18.43 |
| YPPc(g)=yield per plant under control condition; YPPi(g)=yield per plant under infected condition; STI=stress tolerance index; RCI=relative cell injury; CV(%)=cell viability; DI(%)=disease incidence; DSI(%)=disease severity index; RLD(%)=relative leaf damage. | | | | | | | | | |

Supplementary Table S5: Disease incidence and classification of resistance level of 78 chilli genotypes against *Phytophthora capsici* root rot.

| S# | Genotype | DI (%) | RL | S# | Genotype | DI (%) | RL | S# | Genotype | DI (%) | RL |
| --- | --- | --- | --- | --- | --- | --- | --- | --- | --- | --- | --- |
| 1 | 1787 | 99.11 | S | 27 | 32332 | 80 | S | 53 | 1209 | 99.44 | S |
| 2 | 1791 | 100 | S | 28 | 32333 | 83.34 | S | 54 | 9905 | 83.34 | S |
| 3 | 1792 | 80.12 | S | 29 | 32335 | 76.77 | S | 55 | 59328 | 99.33 | S |
| 4 | 1799 | 56.67 | S | 30 | 32336 | 86.67 | S | 56 | Chakwal1 | 83.34 | S |
| 5 | 16162 | 60.33 | S | 31 | 32344 | 100 | S | 57 | Chakwal2 | 50 | S |
| 6 | 16163 | 76.67 | S | 32 | 32350 | 33.34 | MR | 58 | Chakwal3 | 6.81 | R |
| 7 | 16168 | 60.23 | S | 33 | 32351 | 20.22 | MR | 59 | Chakwal4 | 18.86 | R |
| 8 | 16169 | 53.34 | S | 34 | 32354 | 43.44 | MR | 60 | Chakwal5 | 90 | S |
| 9 | 20366 | 99.45 | S | 35 | 32362 | 98.94 | S | 61 | Ghotki | 46.99 | MR |
| 10 | 20372 | 100 | S | 36 | 32370 | 99.5 | S | 62 | Longi | 23.34 | MR |
| 11 | 20451 | 56.13 | S | 37 | 32385 | 100 | S | 63 | PusaJawala | 100 | S |
| 12 | 20523 | 98.78 | S | 38 | 32390 | 100 | S | 64 | Syngenta | 46.77 | MR |
| 13 | 20524 | 100 | S | 39 | 14/9 | 100 | S | 65 | Cdk-101 | 83.34 | S |
| 14 | 24621 | 100 | S | 40 | 16/4 | 100 | S | 66 | Advanta5017 | 14.62 | R |
| 15 | 24623 | 100 | S | 41 | 16/5 | 98.61 | S | 67 | Marvi558 | 100 | S |
| 16 | 24624 | 99.17 | S | 42 | 16/7 | 100 | S | 68 | Greenstar | 99.66 | S |
| 17 | 24625 | 86.91 | S | 43 | 16/8 | 91.73 | S | 69 | Greengold | 99.06 | S |
| 18 | 24626 | 100 | S | 44 | 16/9 | 100 | S | 70 | SuperskyAB | 79.11 | S |
| 19 | 24627 | 98.16 | S | 45 | Skyway | 80 | S | 71 | Kalae542 | 98.71 | S |
| 20 | 24629 | 100 | S | 46 | Skyline2 | 100 | S | 72 | HP-1410 | 99.5 | S |
| 21 | 24634 | 100 | S | 47 | 15/4 | 99.72 | S | 73 | Advanta512 | 100 | S |
| 22 | 32319 | 87.44 | S | 48 | 15/5 | 100 | S | 74 | PH-264 | 100 | S |
| 23 | 32320 | 99.61 | S | 49 | 15/6 | 99.9 | S | 75 | Greenfire | 46.67 | MR |
| 24 | 32321 | 98.68 | S | 50 | KHHP081A | 76.67 | S | 76 | HP1449 | 100 | S |
| 25 | 32324 | 83.34 | S | 51 | 408 | 100 | S | 77 | 1031 | 100 | S |
| 26 | 32328 | 99.56 | S | 52 | 1108 | 100 | S | 78 | Disney | 97.21 | S |
| DI=disease incidence; RL= resistance level; R=resistance; MR=moderately resistance; S=susceptible | | | | | | | | | | | |

Supplementary Table S6: Principal component analysis (PCA) for phenotypic traits using 78 chilli genotypes.

|  | **Principal Component (PC) 1** | **Principal Component (PC) 2** |
| --- | --- | --- |
| Eigen Value | 3.42 | 2.02 |
| Variance (%) | 42.8 | 25.30 |
| Cumulative variance (%) | 42.8 | 68.08 |
| Yield per plant under control (g) | 0.82 | 0.12 |
| Yield per plant under infected (g) | 0.82 | 0.11 |
| Stress tolerance index | 0.86 | 0.07 |
| Relative Cell Injury (%) | 0.11 | 0.04 |
| Cell Viability (%) | 0.44 | 0.02 |
| Disease Incidence (%) | 0.27 | 0.71 |
| Disease Severity Index (%) | 0.09 | 0.54 |
| Relative Leaf Damage (%) | 0.01 | 0.40 |

Supplementary Table S7: Principal co-ordinate analysis (PC0A) of 78 chilli genotypes using 34 SSR markers.

|  | **PCoA 1** | **PCoA 2** |  | **PCoA 1** | **PCoA 2** |  | **PCoA 1** | **PCoA 2** |
| --- | --- | --- | --- | --- | --- | --- | --- | --- |
| **EigenValue** | 9.29 | 6.12 |  |  |  |  |  |  |
| **Variance (%)** | 20.96 | 13.82 |  |  |  |  |  |  |
| **Cumulative variance (%)** | 20.96 | 34.78 |  |  |  |  |  |  |
| **1787** | -0.36 | 0.17 | **32332** | -0.27 | -0.01 | **1209** | 0.32 | 0.22 |
| **1791** | -0.41 | 0.12 | **32333** | -0.03 | -0.34 | **9905** | 0.31 | 0.43 |
| **1792** | -0.46 | -0.12 | **32335** | -0.03 | -0.16 | **59328** | 0.21 | 0.30 |
| **1799** | -0.53 | 0.00 | **32336** | -0.27 | -0.00 | **Chakwal1** | 0.22 | 0.43 |
| **16162** | -0.56 | 0.06 | **32344** | -0.03 | -0.34 | **Chakwal2** | 0.52 | 0.39 |
| **16163** | -0.51 | -0.02 | **32350** | -0.27 | 0.00 | **Chakwal3** | **0.97** | **0.48** |
| **16168** | 0.29 | 0.15 | **32351** | -0.04 | -0.04 | **Chakwal4** | **0.85** | 0.44 |
| **16169** | -0.39 | -0.09 | **32354** | 0.35 | 0.01 | **Chakwal5** | 0.46 | 0.29 |
| **20366** | -0.44 | -0.24 | **32362** | 0.20 | -0.08 | **Ghotki** | 0.20 | 0.44 |
| **20372** | -0.47 | -0.08 | **32370** | 0.24 | -0.01 | **Longi** | 0.18 | 0.41 |
| **20451** | -0.52 | -0.12 | **32385** | 0.17 | 0.02 | **PusaJawala** | 0.15 | 0.45 |
| **20523** | -0.36 | -0.12 | **32390** | -0.06 | -0.09 | **Syngenta** | 0.34 | 0.43 |
| **20524** | -0.38 | 0.04 | **14/9** | -0.26 | 0.17 | **Cdk-101** | 0.14 | **0.47** |
| **24621** | -0.39 | 0.07 | **16/4** | -0.13 | -0.10 | **Advanta5017** | **0.65** | 0.30 |
| **24623** | -0.52 | -0.05 | **16/5** | -0.22 | 0.14 | **Marvi558** | 0.26 | 0.37 |
| **24624** | -0.28 | -0.22 | **16/7** | -0.13 | 0.01 | **Greenstar** | 0.25 | -0.56 |
| **24625** | -0.50 | -0.04 | **16/8** | -0.15 | -0.11 | **Greengold** | 0.39 | -0.32 |
| **24626** | -0.41 | 0.14 | **16/9** | 0.43 | -0.06 | **SuperskyAB** | 0.30 | -0.38 |
| **24627** | -0.15 | 0.26 | **Skyway** | -0.29 | -0.10 | **Kalae542** | 0.29 | -0.48 |
| **24629** | -0.37 | -0.11 | **Skyline2** | -0.01 | 0.20 | **HP1410** | 0.46 | -0.52 |
| **24634** | -0.02 | -0.00 | **15/4** | 0.01 | 0.17 | **Advanta512** | 0.38 | -0.49 |
| **32319** | -0.16 | 0.17 | **15/5** | -0.11 | 0.32 | **PH-264** | 0.33 | -0.64 |
| **32320** | 0.24 | -0.17 | **15/6** | -0.03 | 0.17 | **Greenfire** | 0.33 | -0.52 |
| **32321** | -0.07 | -0.26 | **KHHP081A** | -0.05 | 0.04 | **HP1449** | 0.33 | -0.62 |
| **32324** | -0.20 | 0.07 | **408** | 0.02 | 0.04 | **1031** | 0.36 | -0.49 |
| **32328** | -0.27 | -0.08 | **1108** | -0.24 | 0.13 | **Disney** | 0.33 | -0.36 |

List of Figures

Supplementary Figure S1: Polymorphism among 78 chilli genotypes for SSR markers linked to PcRR resistance in chili a) Hpms1172, b) CAMS177, b) CAMS066, d) CA06g27450 and e) CAMS173. The visually bright bands represents the resistant level, dull bands represents the moderately resistant level and if not then susceptible.


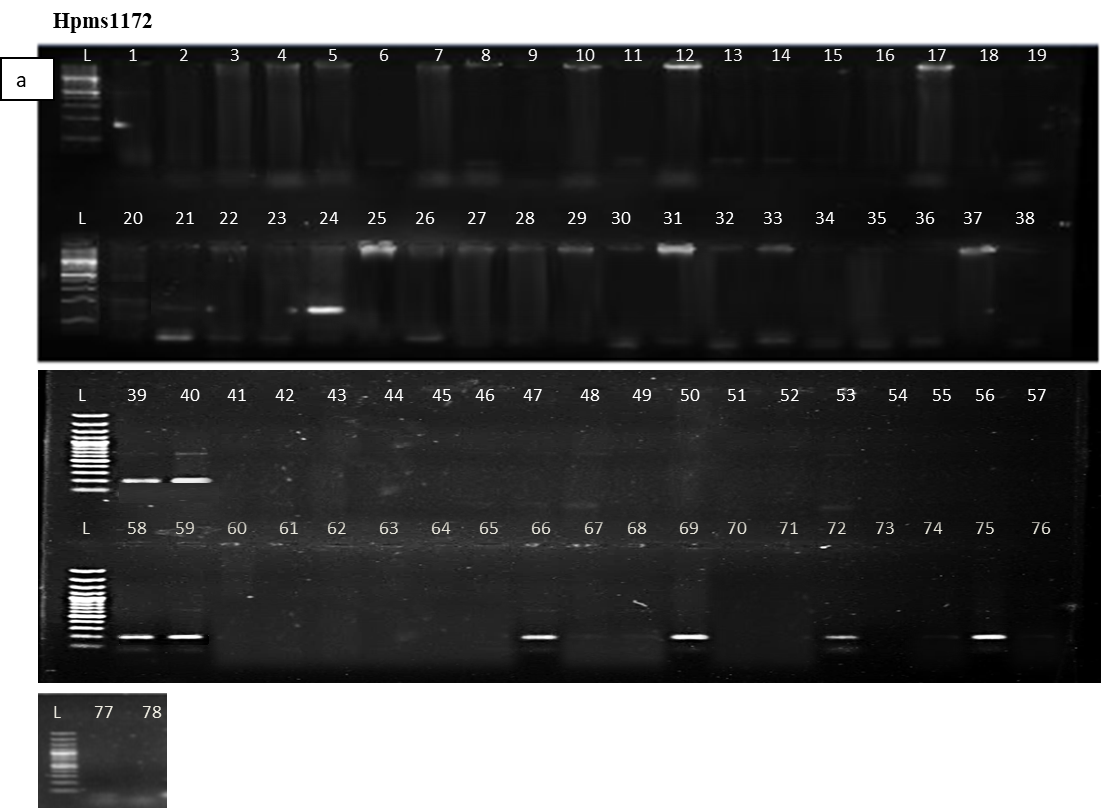


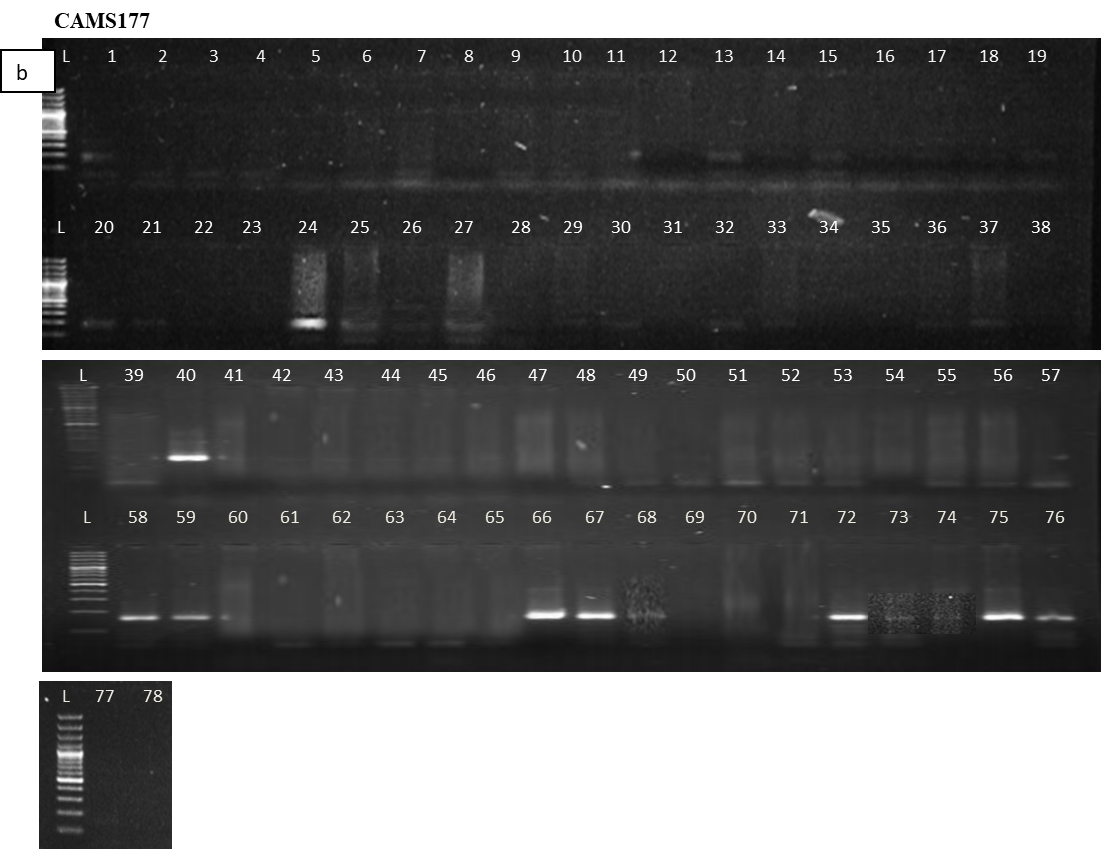


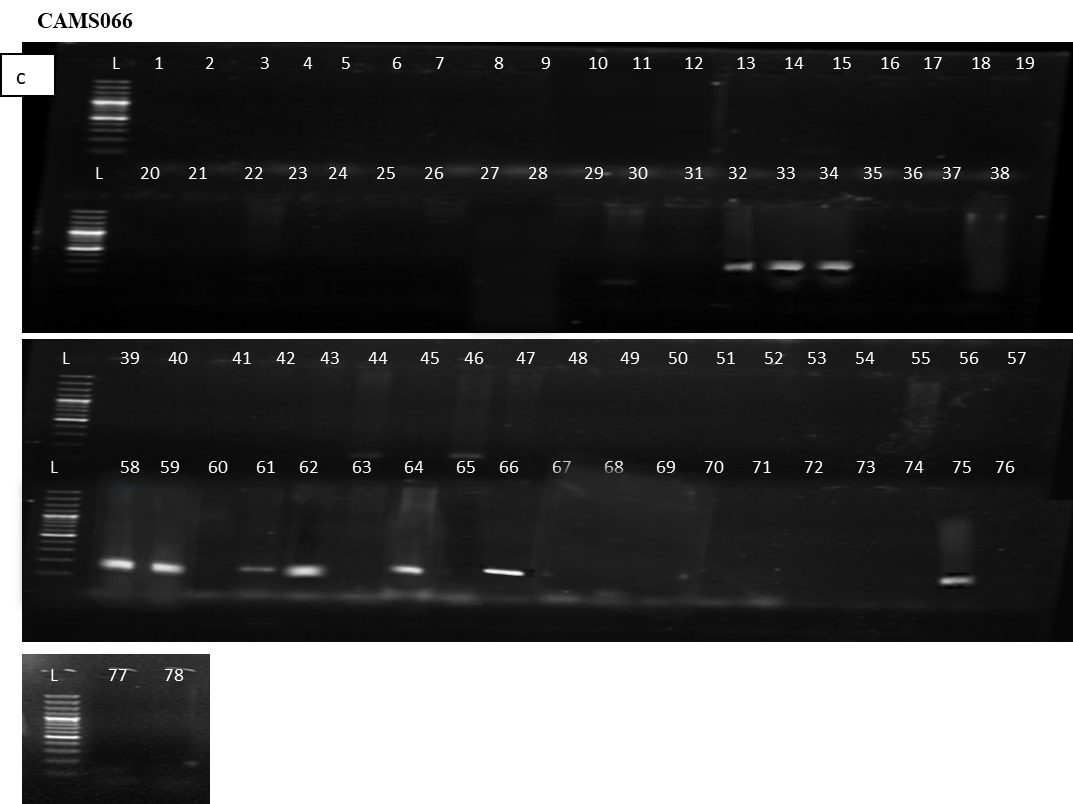


**
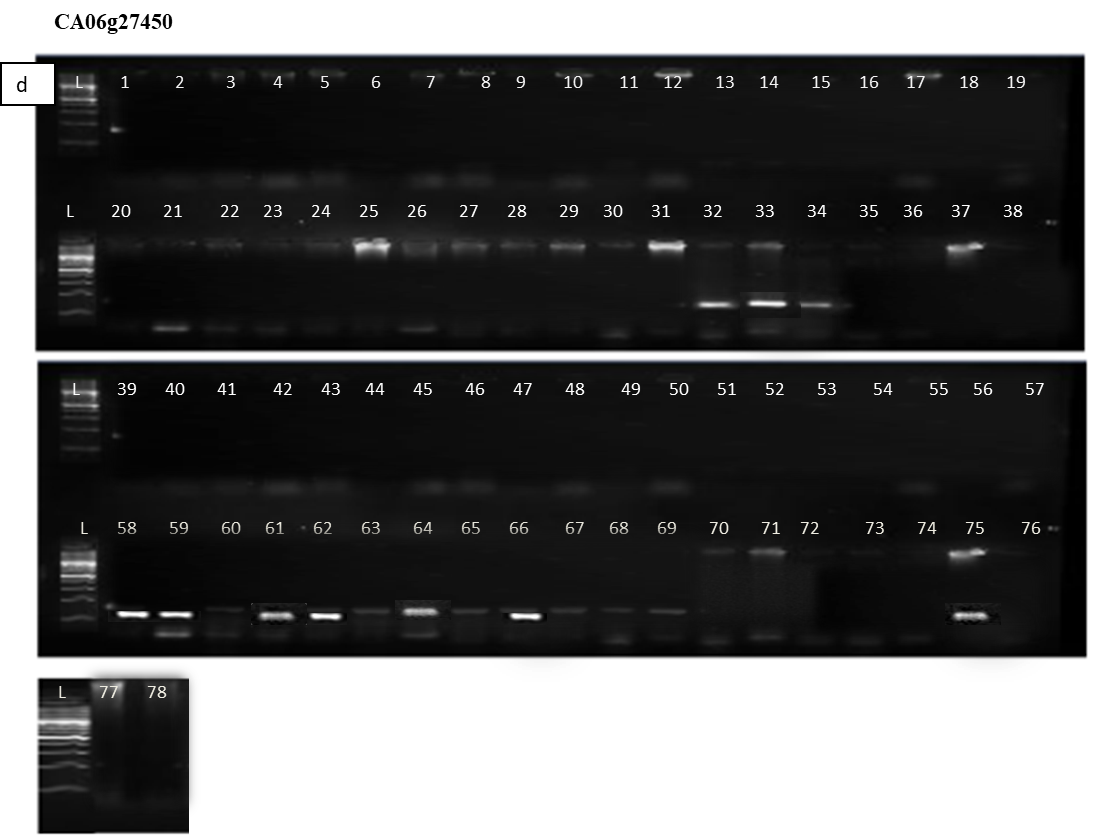
**


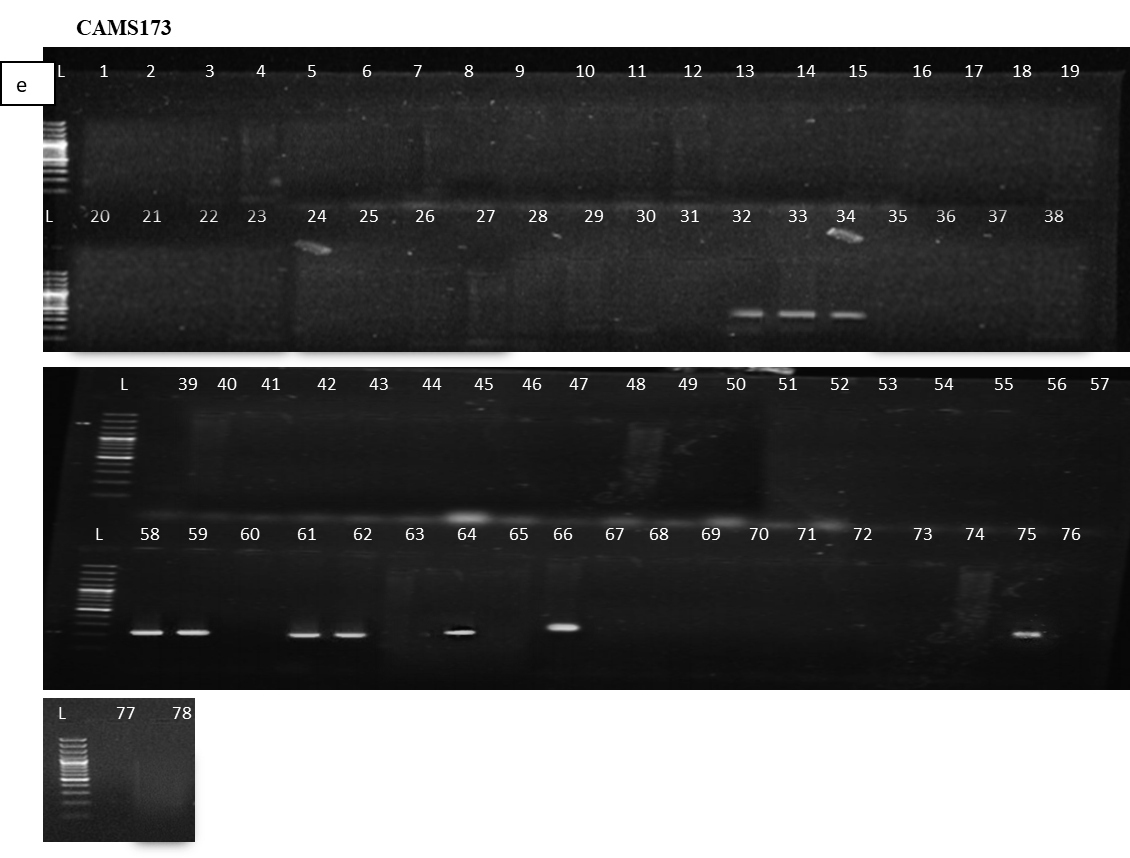


Supplementary Figure S1: Polymorphism among 78 chilli genotypes for SSR markers linked to PcRR resistance in chili a) Hpms1172, b) CAMS177, b) CAMS066, d) CA06g27450 and e) CAMS173. The visually bright bands represents the resistant level, dull bands represents the moderately resistant level and if not then susceptible.
